# PRMT1-dependent methylation of BRCA1 contributes to the epigenetic defense of breast cancer cells against ionizing radiation

**DOI:** 10.1038/s41598-020-70289-3

**Published:** 2020-08-06

**Authors:** María F. Montenegro, Rebeca González-Guerrero, Luis Sánchez-del-Campo, Antonio Piñero-Madrona, Juan Cabezas-Herrera, José Neptuno Rodríguez-López

**Affiliations:** 1 Department of Biochemistry and Molecular Biology A, School of Biology, University of Murcia, Instituto Murciano de Investigación Biosanitaria (IMIB), Murcia, Spain; 2 Department of Surgery, University Hospital Virgen de la Arrixaca, IMIB, Murcia, Spain; 3 Molecular Therapy and Biomarkers Research Group, University Hospital Virgen de la Arrixaca, IMIB, Murcia, Spain

**Keywords:** Cancer, Cell biology

## Abstract

The therapeutic effect of irradiation is thought to come from DNA damage that affects rapidly proliferating cancer cells; however, resistant cells rapidly initiate mechanisms to repair such damage. While DNA repair mechanisms responsible for cancer cell survival following DNA damage are understood, less is known about the epigenetic mechanisms resulting in resistance to radiotherapy. Although changes in DNA methylation are related to mechanisms of long-term resistance, it is more likely that the methylation state of a series of proteins could be responsible for the first-line of defense of cancer cells against irradiation. In this study, we observed that irradiation of breast cancer cells was accompanied by an overproduction in *S*-adenosylmethionine, which increases the activity of cellular methylases. We found that by activating PRMT1, irradiation triggers a BRCA1-dependent program that results in efficient DNA repair and inhibition of apoptosis. Depletion of PRMT1 in irradiated cells resulted in a switch of BRCA1 functions from repair and survival in the nucleus to activation of cell death signals in the cytoplasm. We conclude that by modulating the cellular localization of BRCA1, PRMT1 is an important regulator of the oncogenic functions of BRCA1, contributing to the epigenetic defense of breast cancer cells against ionizing radiation.

## Introduction

Methylation of Lys and Arg residues on non-histone proteins has emerged as a prevalent post-translational modification and as an important regulator of cellular signaling and function^1^. In eukaryotic organisms, arginine methylation is one of the many posttranslational modifications that proteins can undergo^2,3^. This modification is catalyzed by a group of enzymes called protein arginine methyltransferases (PRMTs), which transfer a methyl group from *S*-adenosylmethionine (SAM) to one or two of the guanidino nitrogen atoms in arginine. Of all the PRMTs identified in mammalian cells, PRMT1 is the predominant type I enzyme, being the responsible for at least 85% of all arginine methylation reactions in human cells^4^. In general, PRMT1 prefers substrates with arginines included in glycine- and arginine-rich (GAR) sequences^2^, however, PRMT1 can also methylate substrates that lack a GAR motif, such as PGC1α, estrogen receptor, and FOXO transcription factors^5–7^.

As reflected by the diversity of its substrates, PRMT1 is implicated in the regulation of a plethora of cellular processes^8,9^. For example, arginine methylation by PRMT1 is required for cell proliferation and genome integrity, and it has been reported that losing of PRMT1 in mouse embryonic fibroblasts results in DNA damage, defects in cell cycle checkpoints, aneuploidy, and polyploidy^10^. In addition, PRMT1 is also a key posttranslational modification factor in the DNA damage response (DDR) pathway in proliferating mammalian cells. In this sense, recent investigations demonstrated that methylation of BRCA1 by PRMT1 influenced its transcriptional cofactor functions^11^. In fact, we also observed that a hypomethylating treatment impeded the recruitment of BRCA1 to the chromatin regions flanking double-strand breaks (DSBs), thereby inhibiting DDR signals in breast cancer cells treated with ionizing radiation (IR)^12^. Interestingly, knockdown of PRMT1 resulted in the cytosolic accumulation of BRCA1 after IR and in an increase in susceptibility in MCF7 cells to IR-induced apoptosis. Because BRCA1 needs to be translocated into the nucleus to perform its DNA-repairing functions, these results indicated that methylation of BRCA1 by PRMT1 may be necessary to activate BRCA1-dependent DNA repair in breast cancer cells that are subjected to IR. Whether cytosolic BRCA1 controls apoptotic pathways in breast cancer cells by interacting with antiapoptotic proteins such as Bcl-2 is also an issue to be resolved^13,14^.

In response to radiation, cancer cells activate multiple stress signals. Although cancer cells tend to convert glucose into lactate (the Warburg effect), it has been recently discovered that, after radiation, tumor cells quickly relocated mTOR to mitochondria, which was accompanied by a decreased in lactate production and a substantial increase of mitochondrial ATP formation and oxygen consumption^15^. The results indicated that tumor cells could quickly react to genotoxic stress by an mTOR-dependent pathway that reprogram the bioenergetics of the cells switching from aerobic glycolysis to mitochondrial oxidative phosphorylation. In addition to this metabolic change, epigenetic events have been shown to be crucial for damage repair and survival after exposure to IR. For instance, we described that a hypomethylating treatment designed to disturb the metabolism of adenosine by dipyridamole in the presence of 3-*O*-(3,4,5-trimethoxybenzoyl)-(–)-catechin, a synthetic antifolate, efficiently sensitized breast cancer cells to radiotherapy^12^. This treatment highly reduced the self-renewal capability of breast cancer stem cells and reverted the mesenchymal phenotype of breast cancer cells. The metabolic reprogramming that occurs in response to the cellular environment has been shown to regulate complex metabolic processes, such as osteoclast differentiation, through the modulation of specific methylases^16^. Therefore, understanding the possible connection between metabolic and epigenetic events that result in the resistance of breast cancer cells to IR was the main goal of this research. In this context, we identified PRMT1 as a methyltransferase that contributes to the epigenetic defense of cancer cells against ionizing radiation. These results could have clinical implications because we identified a targeted epigenetic network that controls not only DNA repair mechanisms but is also connected to suppressing apoptotic pathways in response to radiotherapy.

## Results

### IR induces PRMT1-dependent methylation of BRCA1 in breast cancer cells

**Figure 1:**
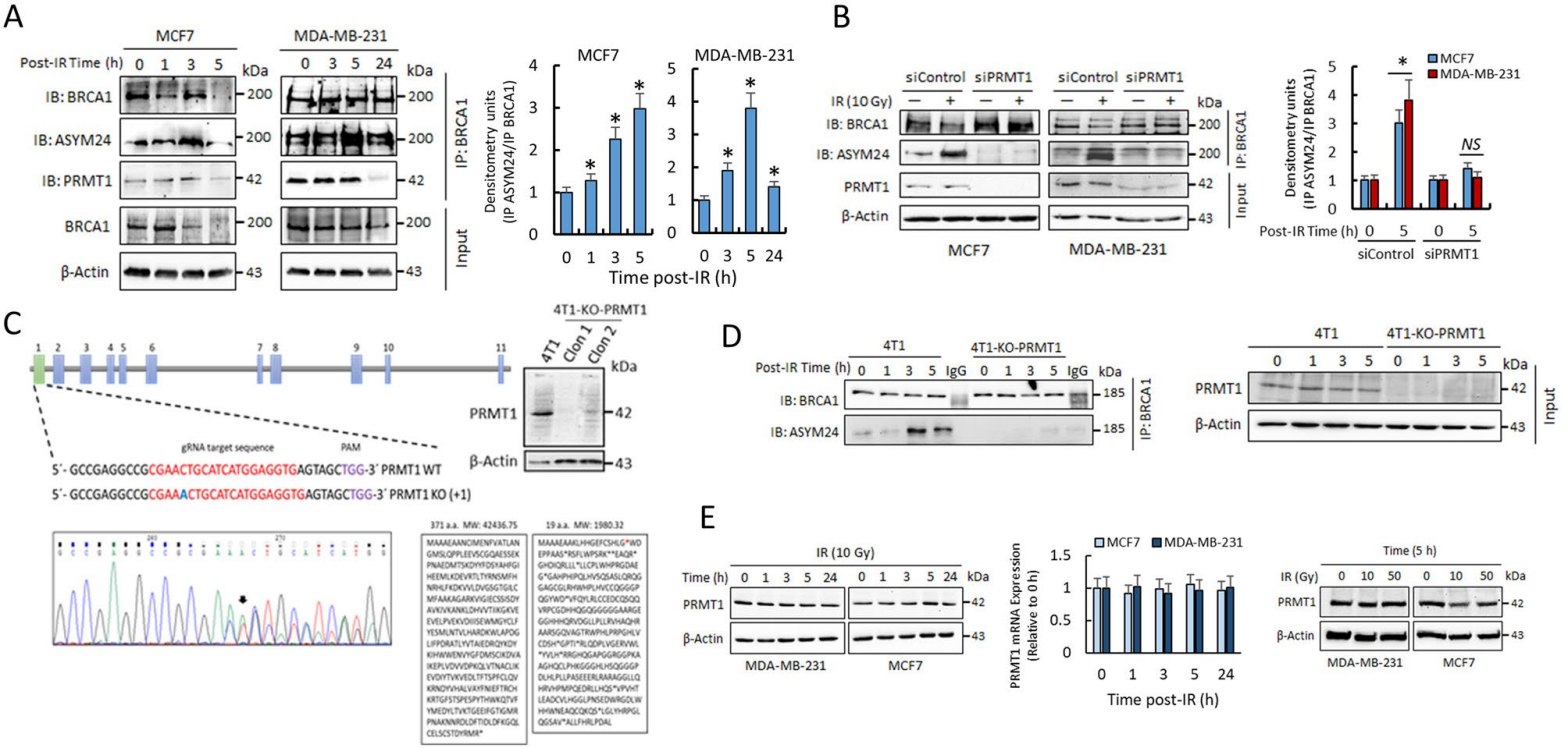
IR induces PRMT1-dependent methylation of BRCA1. (**A**) Methylation status of BRCA1 in breast cancer cells after IR treatments (10 Gy). One milligram of cytosolic extracts was immunoprecipitated using an anti-BRCA1 antibody (clone 6B4), separated using SDS-PAGE and immunoblotted with the indicated antibodies. The histogram represents methylated BRCA1 (IP ASYM24) with respect to immunoprecipitated BRCA1 (IP BRCA1) at different post-IR times and relative to nonirradiated cells after correction by the amount of β-actin. The IR-dependent increase in BRCA1 methylation was statistically significant when compared with nonirradiated cells (*P < 0.005). (**B**) Effect of PRMT1 silencing on BRCA1 methylation in breast cancer cells. The histogram represents methylated BRCA1 (IP ASYM24) with respect to immunoprecipitated BRCA1 (IP BRCA1) relative to nonirradiated cells and β-actin. The IR-dependent increase in BRCA1 methylation was statistically significant when compared with nonirradiated cells in siControl cells (*P < 0.005) but not significant (*NS*) in siPRMT1 cells. (**C**) PRMT1 gene knockout using the CRISPR/Cas9 system in 4T1 cells. The schematic diagram shows the guide RNA (gRNA) targeting site on exon 1 of the PRMT1 gene. Protospacer adjacent motif (PAM) sequences are also presented. The figure also shows Sanger sequencing analysis of PCR fragments amplified from gRNA target regions (the inserted nucleotide is in blue) and protein sequence in wild type (WT) and knockout (KO) cells (PRMT1 protein expression in WT and two selected clones was assayed by western blot). (D) Methylation status of BRCA1 in 4T1 and 4T1-KO-PRMT1 cells. Conditions were as indicated in section A of this figure. (**E**) Time- and dose-dependent effects of IR on PRMT1 expression at the protein (western blots) and mRNA (histogram) levels in MDA-MB-231 and MCF7 cells. The groupings blots in this figure were cropped from different gels. Full blots are shown in the Supplementary Information, Fig. S5.

Because the cellular localization and functions of BRCA1 seem to be controlled by protein methylation^11,12^, we sought to determine whether IR modulated the methylated status of this protein. To concentrate on the specific cytosolic modifications of BRCA1 and to avoid interferences by nuclear BRCA1, the methylation status of this protein was analyzed in post-nuclear fractions (referred to as cytosolic fractions). As observed in Fig. 1A, IR induced transient methylation of BRCA1 in both MCF7 and MDA-MB-231 cell lines, as determined after immunoprecipitation of BRCA1 and blotting with both anti-BRCA1 and an antibody that specifically recognized asymetrically dimethylated BRCA1 (ASYM24; Supplementary Fig. S1 and Fig. S10). BRCA1 was detected as a double electrophoretic band at approximately 220 and 189 kDa, corresponding to the lower band of methylated BRCA1. The specificity of BRCA1 immunoprecipitation was evaluated by immunoprecipitation with mouse IgG and with experiments in siBRCA1 MCF7 cells (Supplementary Fig. S1 and Fig. S10). Since PRMT1 coimmunoprecipitated with BRCA1 (Fig. 1A), whether methylation of BRCA1 after IR was dependent on PRMT1 was confirmed by immunoprecipitation assays in MCF7 and MDA-MB-231 cells. In both cases, silencing of PRMT1 resulted in reduced BRCA1 methylation after IR treatments (Fig. 1B). Similar results were obtained from experiments carried out with a 4T1 murine breast cancer cell line in which *PRMT1* was efficiently knocked out using CRISPR/Cas9 (Fig. 1C,D).

### IR increases SAM production in breast cancer cells

**Figure 2:**
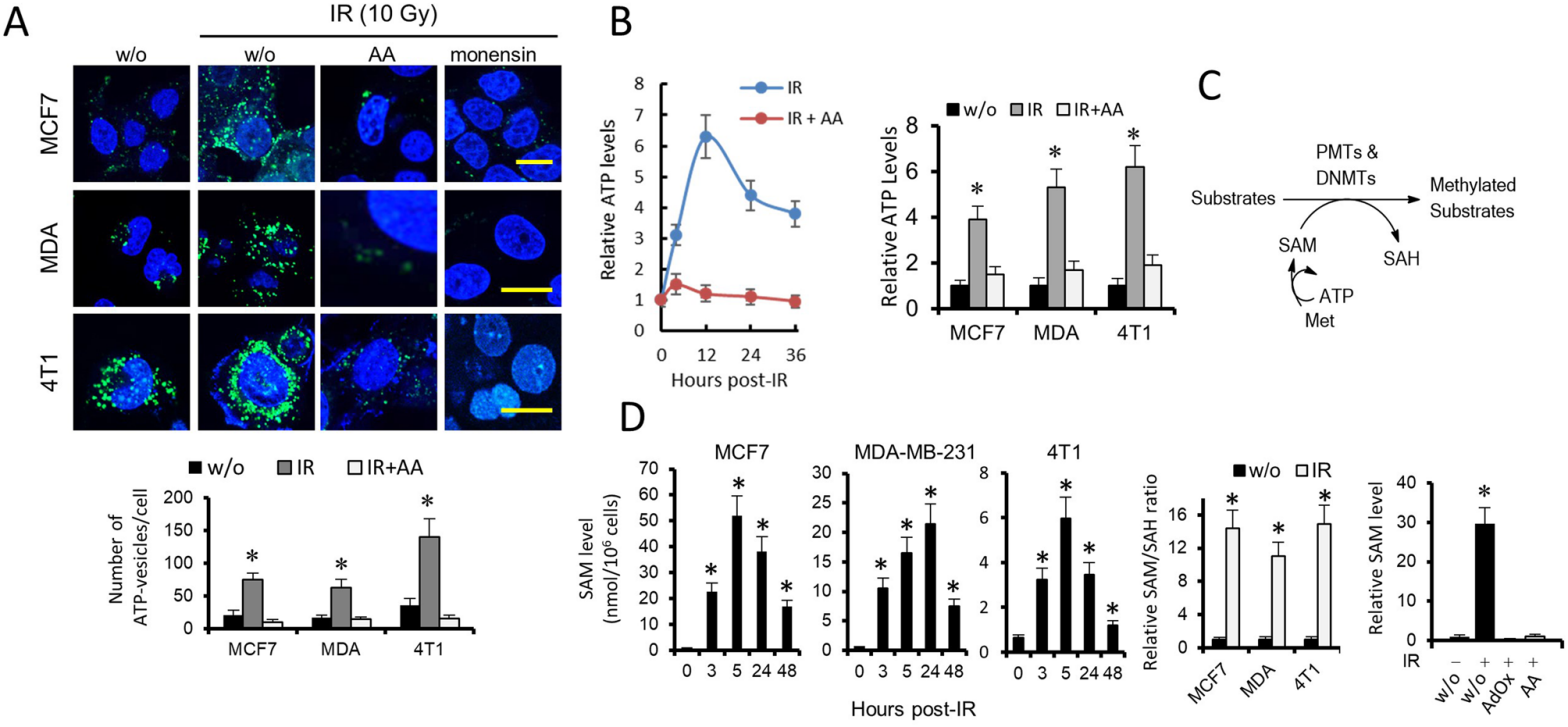
IR increases cellular methylase activity through its coupling to a SAM-producing metabolic pathway. (**A**) Effects of IR, the respiratory chain inhibitor AA and monensin (10 µM) on quinacrine-stained breast cancer cells. Cells were loaded with 10 µM quinacrine. IR led to the accumulation of quinacrine fluorescence in breast cancer cells, whereas AA and monensin led to the loss of quinacrine fluorescence. w/o, without treatment. Scale bars refer to 10 µm. (**B**) The histogram represents the effects of IR (10 Gy, 12 h) and/or AA on ATP levels in breast cancer cells. *P < 0.05. Time-course (left panel) was determined using 4T1 cells. (**C**) The methionine metabolic pathway coupled to epigenetic regulation. (**D**) Effect of IR (10 Gy) on the methylation capacity of breast cancer cells. The increases in SAM and SAM/SAH ratios were statistically significant after IR (*P < 0.05) when compared with control (w/o) samples. AA and AdOx led to a loss of SAM increase in MDA-MB-231 cells after IR (10 Gy; 5 h) (*P < 0.05).

To understand the molecular mechanism by which IR induced PRMT1-dependent methylation of BRCA1 in breast cancer cells, we first analyzed the effect of IR on PRMT1 expression at both the protein and mRNA levels. Unexpectedly, we found no significant time-dependent changes in the expression of PRMT1 in cells subjected to 10 Gy of radiation or in cells subjected to higher doses of radiation (Fig. 1E). Therefore, we next examined possible metabolic changes associated with the IR treatments. Breast cancer cells grown under control conditions and stained with the ATP-sensitive fluorochrome quinacrine (Fig. 2A), which is typically used for the detection of intracellular ATP-containing vesicles, emits green fluorescence that can be monitored by cytofluorometry. This signal was significantly augmented when cells were subjected to IR but attenuated when control or irradiated cells were treated with antimycin A (AA; Fig. 2A), which inhibits mitochondrial respiration at the level of complex III. The specificity of ATP-quinacrine signals was confirmed with experiments in the presence of monensin, a negative control that causes a decrease in the fluorescence of quinacrine puncta (Fig. 2A). Determination of ATP in breast cancer cell extracts by a luciferase-based assay indicated that IR induced a transient increase in intracellular ATP stores, which was also mitigated by the presence of AA (Fig. 2B). Overall, the results indicated that irradiation of breast cancer cells induced a metabolic shift toward oxidative metabolism, which was accompanied by an increase in ATP production.

Although epigenetic regulation is known to be a fundamental mechanism for various cellular processes, the epigenetic factors mediating the response of cancer cells to IR are less known. The SAM/*S*-adenosylhomocysteine (SAH) ratio is regarded as an indicator of cellular methylation capacity, and an increase in this ratio may predict a higher cellular methylation status. Since intracellular ATP is coupled to the methionine pathway to control epigenetic regulation (Fig. 2C)^16^, we next investigated whether IR-induced mitochondrial ATP production resulted in SAM upregulation. Therefore, we analyzed the time course of SAM/SAH ratios in a variety of breast cancer cells after exposure to IR (Fig. 2D). Although IR differentially modulated the SAH content in the analyzed cells, we observed a consistent and significant increase in intracellular SAM (peaked at 5–24 h), which shifted the SAM/SAH ratio toward a higher methylation status in all cancer cell lines (Fig. 2D). As expected, inhibition of SAH hydrolase by adenosine-2,3-dialdehyde (AdOx) impeded SAM accumulation in irradiated cells (Fig. 2D). Treatment of breast cancer cells with AA also prevented the overproduction of SAM after IR (Fig. 2D), which indicated a functional link between the metabolic adaptation of breast cancer cells to radiation and the control of epigenetic events.

### IR increases cellular methylase activity through its coupling to a SAM-producing metabolic pathway

**Figure 3:**
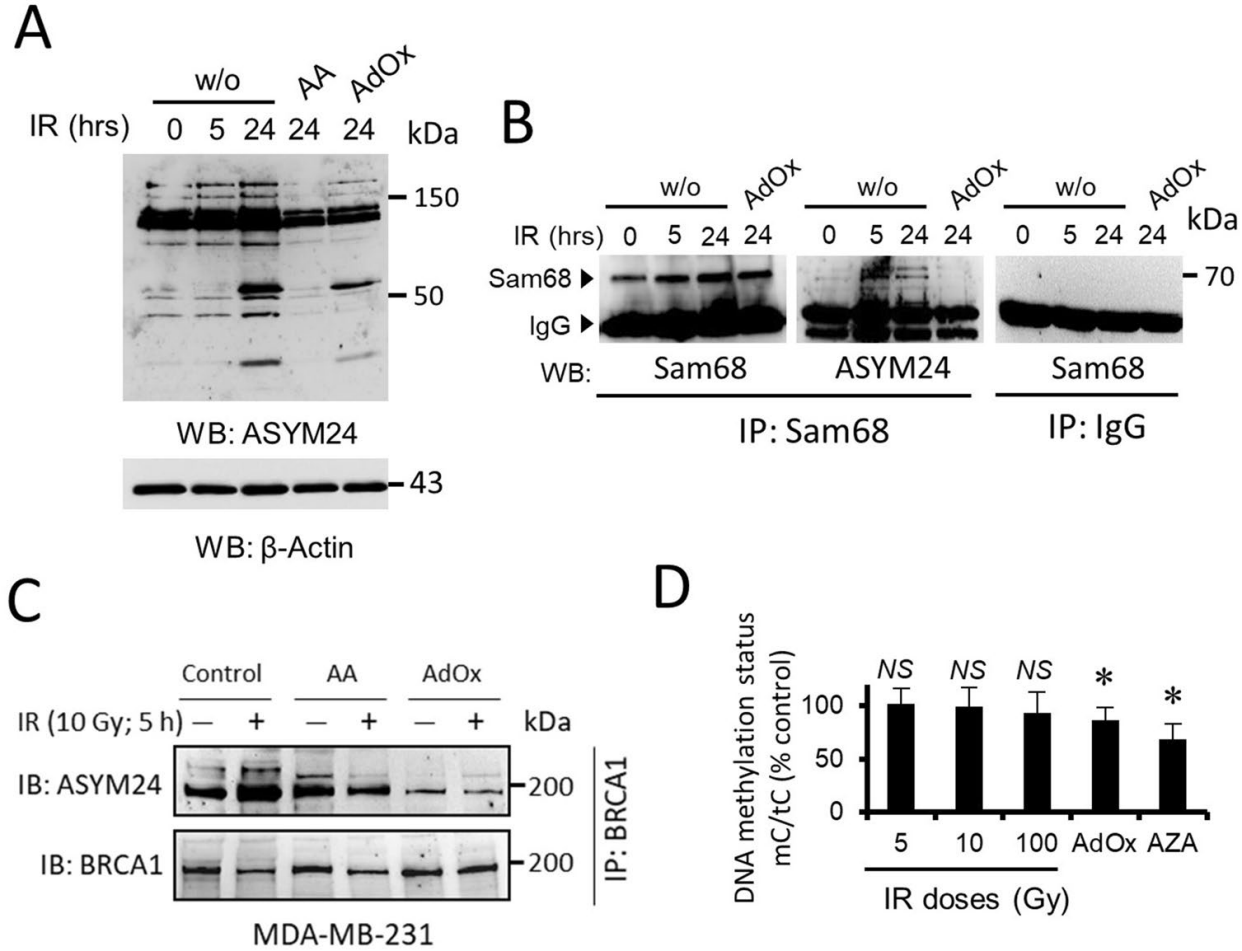
IR induces hypermethylation of cellular proteins, but not DNA hypermethylation, in MDA-MB-231 cells. (**A**) Whole-cell lysates subjected to specified conditions (IR, 10 Gy) were immunoblotted with anti-asymmetrical dimethylarginine (ASYM24; full western blot) and anti-β-actin antibodies to control for equivalent loading. (**B**) Whole-cell lysates were immunoprecipitated with anti-Sam68 antibodies. The bound proteins were separated by SDS-polyacrylamide gel electrophoresis and immunoblotted with anti-Sam68 and ASYM24 antibodies as indicated. A control experiment in which cell lysates were immunoprecipitated with IgG is shown. (**C**) Effect of AA and AdOx on IR-induced methylation of BRCA1. MDA-MB-231 whole-cell lysates were immunoprecipitated with anti-BRCA1 antibody and immunoblotted with anti-ASYM24 antibody. Equal amounts of BRCA1 per lane were loaded. (**D**) Effect of IR (24 h), AdOx and AZA (5 µM) treatment on the genomic DNA methylation status in MDA-MB-231 cells, evaluated as the ratio of 5-methylcytosine/total cytosine (mC/tC). The histogram represents the means ± SD of three independent experiments. Statistical significance (*P*) was determined using Student’s paired *t* test, *P < 0.05 and *NS* (not significant) with respect to the untreated control. Except for those indicated, the groupings blots in this figure were cropped from different gels. Full blots are shown in the Supplementary Information, Fig. S6.

Next, we aimed to determine the extent to which the protein methylation status was affected by the accumulation of intracellular SAM. For this, we immunoblotted total cellular extracts of MDA-MB-231 cells with a methyl-arginine-specific antibody. Irradiation of MDA-MB-231 cells resulted in hypermethylation of many cellular proteins, as detected with anti-ASYM24 antibody (Fig. 3A). This effect was greatly attenuated when cells were pretreated with AdOx or AA before IR (Fig. 3A). To further confirm the impact of SAM accumulation, we immunoprecipitated a previously defined PRMT1 substrate, Sam68^17^, and examined its methylation status. The indicated samples were immunoprecipitated with an anti-Sam68 antibody and immunoblotted with either anti-Sam68 as a control or anti-ASYM24 antibody to monitor methylation. The hypermethylation of Sam68 was clearly visible when compared with that in untreated or AdOx-pretreated cells (Fig. 3B). Similar inhibitory effects of AdOx and AA were observed when we tested the effect of IR on the asymmetric dimethylation status of BRCA1 in MDA-MB-231 cells (Fig. 3C).

In addition to arginine methylation, we investigated whether SAM production after IR might also affect the activity of other methylases within the cells. For this, we analyzed the methylation status of PP2A, a trimeric serine/threonine phosphatase that contains regulatory subunit B, which is recruited by a C-A dimer composed of catalytic subunit C (PP2A-C) and structural subunit A. Recruitment occurs when C is carboxyl-methylated on the terminal Leu309, resulting in the assembly of the active PP2A trimer^18^. Leucine carboxyl methyltransferase (LCMT-1), a specific SAM-dependent enzyme, catalyzes the methylation of PP2A, and here, we observed that irradiation of breast cancer cells induced IR-dependent methylation of PP2A, which resulted in the catalytic activation of this phosphatase (Supplementary Fig. S2 and Fig. S11).

### Protein methylation is more sensitive to IR-induced SAM accumulation than DNA methylation

Contrary to its effect on protein methylation, intracellular SAM accumulation had a lesser impact on global DNA methylation. The DNA methylation status of MDA-MB-231 cells, defined by the ratio of 5-methylcytosine to total cytosine in DNA hydrolysates, decreased by a nonsignificant 7% (*P* = 0.033) under the highest dose of IR, whereas no effect was observed with lower (5 and 10 Gy) IR doses (Fig. 3D). Accordingly, with data that suggested that DNA methylation was poorly affected by fluctuations in SAH levels^19^, here, we found that inhibition of SAH hydroxylase by AdOx, which induces SAH accumulation, showed small changes in DNA methylation (a decrease of 13%; *P* < 0.01) (Fig. 3D). In contrast, and as expected, incubation with 5-aza-2′-deoxycytidine (AZA), a specific inhibitor of DNA methyltransferases (DNMTs), resulted in a significant 32% decrease (*P* < 0.001) in the 5-methylcytosine to cytosine ratio (Fig. 3D). Altogether, the results indicated that arginine methylation is more sensitive to SAM accumulation than DNA methylation and suggested that protein methylation could be the first line of epigenetic defense of breast cancer cells against IR.

### The cellular localization of BRCA1 is epigenetically controlled by PRMT1

**Figure 4:**
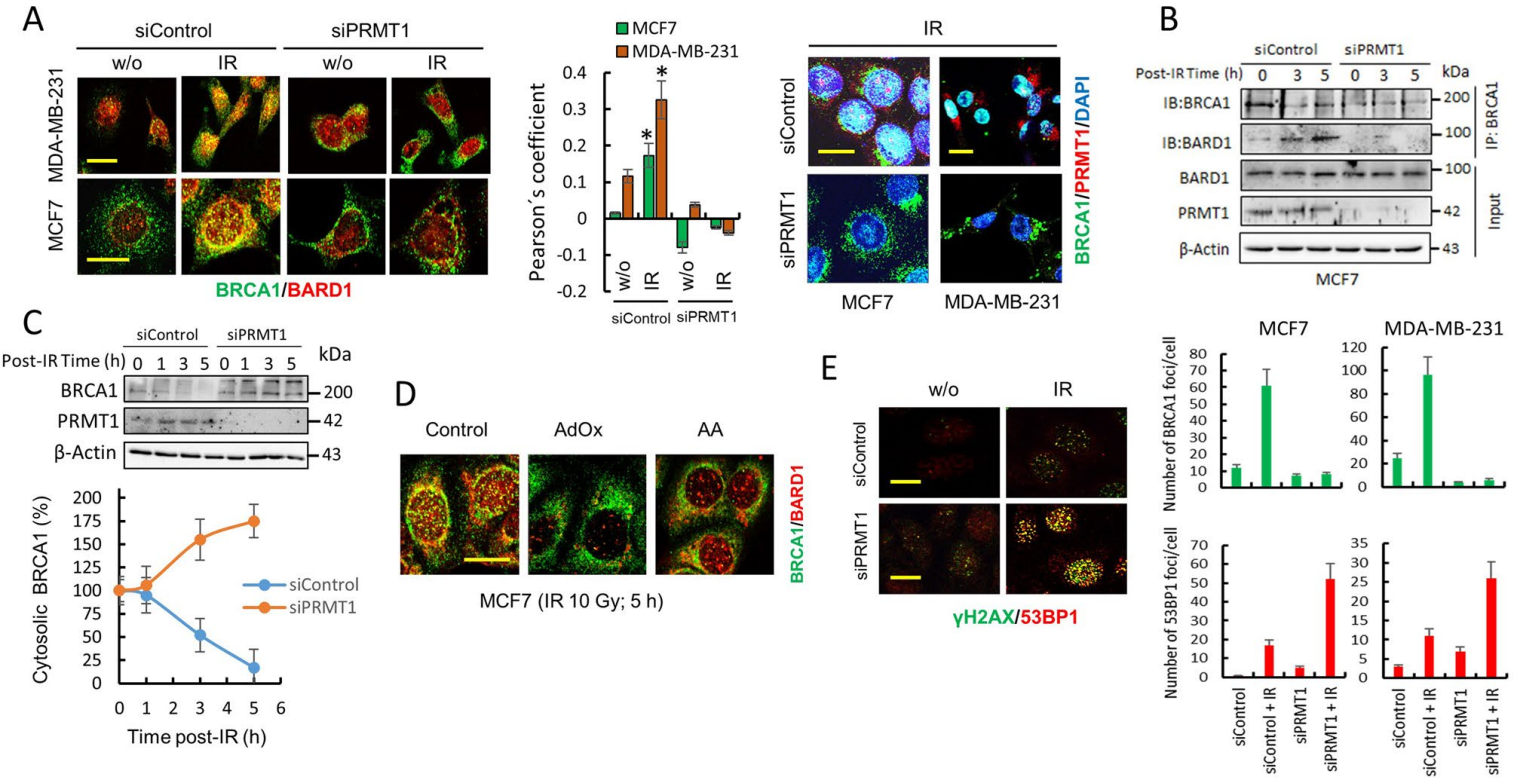
PRMT1 controls the cellular localization of BRCA1 and facilitates DNA homologous recombination. (**A**) The effect of IR (10 Gy) on the localization of BRCA1 and BARD1 in controls and PRMT1-depleted breast cancer cells. Analysis of these proteins was performed using confocal microscopy at 5 h post IR. From the confocal images, a Pearson coefficient was calculated to estimate the degree of colocalization of BRCA1 (clone MS110) with BARD1 (histogram). The Pearson overlap coefficients are represented as the average of 10 individual cells. *P < 0.05 with respect to untreated control cells. The scale bars (10 μm) refer to both panels. Efficient PRMT1 silencing is observed in the right panel (Bars, 15 μm). (**B**) BRCA1 and BARD1 interaction after IR treatments (10 Gy) was determined by immunoprecipitation assays. Total extracts of MCF7 were immunoprecipitated with an anti-BRCA1 antibody (clone 6B4) and immunoblotted with indicated antibodies. (**C**) Time course of cytosolic BRCA1 in siControl- and siPRMT1-transfected MCF7 cells after IR (10 Gy). Cytosolic lysates (20 µg of total protein per well) were separated using SDS-PAGE and immunoblotted with the indicated antibodies. Cytosolic BRCA1 in western blot membranes was calculated by densitometry and referred to cytosolic BRCA1 in non-irradiated controls (100%) after correction by the amount of β-actin. Error bars show mean ± SD of three independent experiments. (**D**) The effect of AA and AdOx on BRCA1/BARD1 foci formation in MCF7 cells. Scale bar, 10 μm. (**E**) The effect of IR (10 Gy; 5 h) on the localization of γH2AX and 53BP1 in controls and PRMT1-depleted MCF7 cells. The histograms demonstrate the quantification of the cells that were positive for BRCA1 (from **A**) and 53BP1 foci under specified conditions. Cells with positive BRCA1 and 53BP1 foci were evaluated in at least 10 fields at × 960 magnification. Scale bars, 10 μm. The groupings blots in this figure were cropped from different gels. Full blots are shown in the Supplementary Information, Fig. S7.

BRCA1 is a nuclear-cytoplasmic shuttling protein, and its function may be regulated via active shuttling between cellular compartments^20^. When in the nucleus, BRCA1 controls high fidelity repair of damaged DNA, but in contrast, BRCA1 has been shown to enhance p53-independent apoptosis when cytoplasmic^20^. BARD1 is a critical regulator of BRCA1 subcellular localization, and it is well established that BARD1 induces BRCA1 intranuclear foci formation by increasing RING-dependent BRCA1 nuclear import and inhibiting BRCA1 nuclear export^21^. Therefore, the consequences of PRMT1 silencing on the co-localization and co-immunoprecipitation of BRCA1 with BARD1 after IR were analyzed using appropriated techniques. Immunoprecipitation and confocal microscopy assays indicated that PRMT1 activity was necessary for BRCA1/BARD1 interaction and for the generation of BRCA1/BARD1 nuclear foci after IR (Fig. 4A,B). In fact, in PRMT1-silenced breast cancer cells, BRCA1 did not accumulate in the nucleus after IR (Fig. 4A). In agreement with the confocal microscopy experiments, which showed rapid translocation of BRCA1 to the nucleus in response to IR, we observed a consistent time-dependent reduction in cytoplasmic BRCA1 in control MCF7 cells after IR; however, silencing of PRMT1 in these cells did not deplete cytosolic BRCA1 after irradiation (Fig. 4C). Other treatments designed to prevent SAM or ATP accumulation in MCF7 cells also impeded BRCA1/BARD1 nuclear foci formation after IR (Fig. 4D). Altogether, the results indicated that PRMT1 controls BRCA1 localization after IR.

### PRMT1 facilitates BRCA1-mediated DNA homologous recombination repair and contributes to DSB repair in breast cancer cells after IR

BRCA1 and 53BP1, two well-defined substrates of PRMT1, play a decisive role in DNA DSB repair mechanisms. While 53BP1 inhibits end resection and facilitates non-homologous end-joining primarily during the G1 phase of the cell cycle, BRCA1 promotes DNA end resection and homologous recombination during the S/G2 phases^22,23^. This competitive relationship is critical for genome integrity during cell division. Because BRCA1 needs to be translocated into the nucleus to perform its DNA-repairing functions, we next analyzed the effects of the loss of PRMT1 on DNA repair after IR. Dysfunction of BRCA1-mediated DNA repair in PRMT1-silenced breast cancer cells was also evidenced by monitoring 53BP1 foci formation in irradiated cells (Fig. 4E). As observed in Fig. 4A,E, the presence of active PRMT1 favored the formation of BRCA1 foci after IR, whereas the absence of this methylase induced the accumulation of 53BP1 foci in breast cancer cells. Since the NHEJ-promoting function of 53BP1 is inhibited by BRCA1, loss of BRCA1 at DSBs in PRMT1-silenced breast cancer cells may suppress HR and direct DNA repair toward NHEJ. The available evidence clearly shows that the balance of BRCA1 and 53BP1 controls the choice of DNA repair pathway and that this balance is cell cycle regulated. Since 53BP1-initiated NHEJ repair is confined to the G1 phase, we next examined the impact of silencing PRMT1 on the cell cycle distribution of MDA-MB-231 cells after IR treatments (Supplementary Fig. S3). Although control cells predominantly accumulated in the G2/M phase of the cell cycle at 24 h post IR, PRMT1-knockdown cells were more homogeneously distributed in the three stages of the cell cycle, with a significant increase in cells accumulated in the G0/G1 and S phases compared with the irradiated control cells. Taken together, these data indicate that PRMT1-dependent methylation of BRCA1 is necessary for efficient DNA HR repair^24^.

### PRMT1-dependent methylation of BRCA1 contributes to the epigenetic defense of breast cancer cells against IR

**Figure 5:**
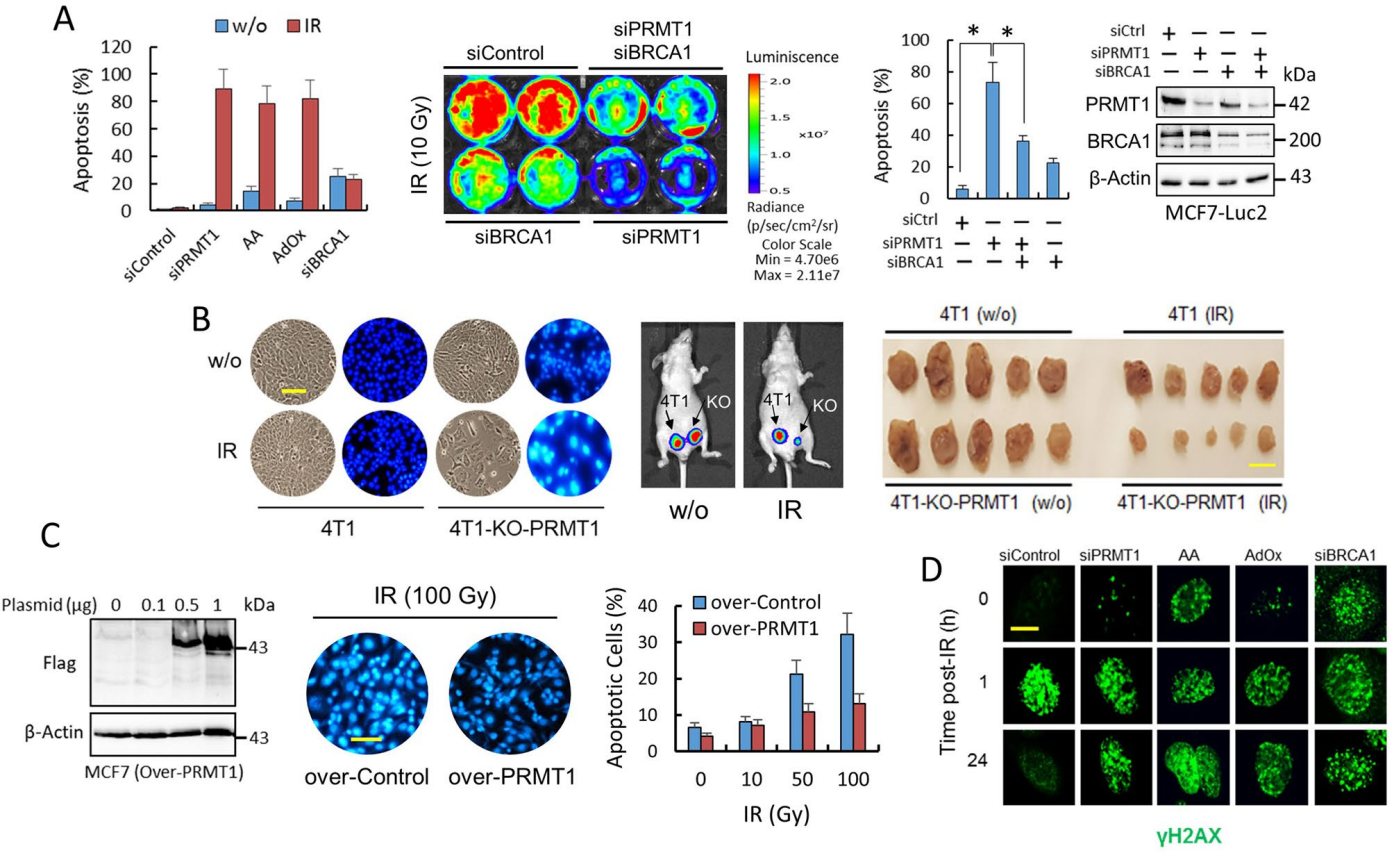
PRMT1 protects breast cancer cells from IR-induced apoptosis. (**A**) Apoptosis determination in MCF7 cells subjected to the indicated treatments using an ELISA (left panel). At the zero time point, the cells were treated with AA and AdOx. On day 1, the cells were irradiated (10 Gy), and apoptosis was assayed on day 4. When the cells were not irradiated, apoptosis analyses were performed on the fourth day. In the right panel, MCF7-Luc2 cells were subjected to indicated siRNA transfections. The number of cells remaining at three days post-IR was quantified by bioluminescence imaging, while apoptosis was determined by ELISA (histogram; *P < 0.05). (**B**) Depletion of PRMT1 sensitizes 4T1 cells to IR in vitro and in vivo. The images in the left panel show the effect of IR on apoptosis in the indicated 4T1 cells. The scale bar (100 μm) refers to both panels. 4T1-control-transfected and 4T1-PRMT1-KO cells were subcutaneously injected near the mammary fat pads of athymic nu/nu mice (arrows represent the initial injection site). Primary tumors were recorded by using bioluminescence imaging (n = 5 per group) and compare nonirradiated (w/o) vs. IR-treated mice (middle panel). Right panel shows the tumor sizes at 40 days after the tumor cells were injected (scale bar, 1 cm). The growth of 4T1-PRMT1-KO tumors in IR-treated mice was significantly reduced (*P* < 0.005) compared with the other groups. (**C**) Overexpression of PRMT1 protects MCF7 cells from IR-induced apoptosis. Western blots showing PRMT1 expression (left panel). The images show the effect of IR on apoptosis in MCF7 cells using Hoechst 33342. Analyses of apoptosis were performed at 72 h after IR. The scale bar (100 μm) refers to both panels. (**D**) Nuclear focus formation of γH2AX in MCF7 cells was observed following double-stranded DNA damage after the indicated treatments. Scale bar represents 8 μm. Blots in this figure were cropped from different gels. Full blots are shown in the Supplementary Information, Fig. S8.

Although it is well recognized that sequestration of BRCA1 away from the nucleus might switch BRCA1 function from DNA repair in the nucleus to activation of cell death signals in the cytoplasm^13^, the mechanisms by which cytoplasmic BRCA1 induces apoptosis are not completely understood. Since PRMT1, through BRCA1 methylation, controls the localization of BRCA1, we next examined whether PRMT1 protects breast cancer cells from IR-induced apoptosis. We observed that PRMT1 depletion or treatments designed to prevent SAM or ATP accumulation in breast cancer cells made the cells more susceptible to IR-induced apoptosis both in vitro and in vivo (Fig. 5A,B), while PRMT1 overexpression protected MDA-MB-231 cells from apoptosis at high IR doses (Fig. 5C). Although we observed a good correlation between the lack of BRCA1 in the nucleus and the inability of cells to repair their DNA (as indicated by the continuous activation of γH2AX in nuclear foci) (Fig. 5D), we observed that cytosolic BRCA1 was needed for efficient IR-induced apoptosis (Fig. 5A,D). Silencing of BRCA1 resulted in constitutive γH2AX foci activation and apoptosis in MCF7 cells; however, IR did not have an additional effect on the constitutive apoptosis induced by BRCA1 depletion (Fig. 5A,D). To assess the role of BRCA1 in PRMT1-dependent IR sensitivity, we have knockdown PRMT1 in the absence of BRCA1. Although PRMT1 sensitized MCF7-Luc2 cells to IR-induced apoptosis, double silencing of PRMT1 and BRCA1 resulted in increased resistance to IR (Fig. 5A). Altogether, these results indicated that PRMT1 is an important component of the epigenetic defense of breast cancer cells against IR and that cytosolic BRCA1 is required for IR-induced apoptosis in breast cancer cells.

### PRMT1, through the methylation of BRCA1, modulates the stability and location of Bcl-2 in breast cancer cells

**Figure 6:**
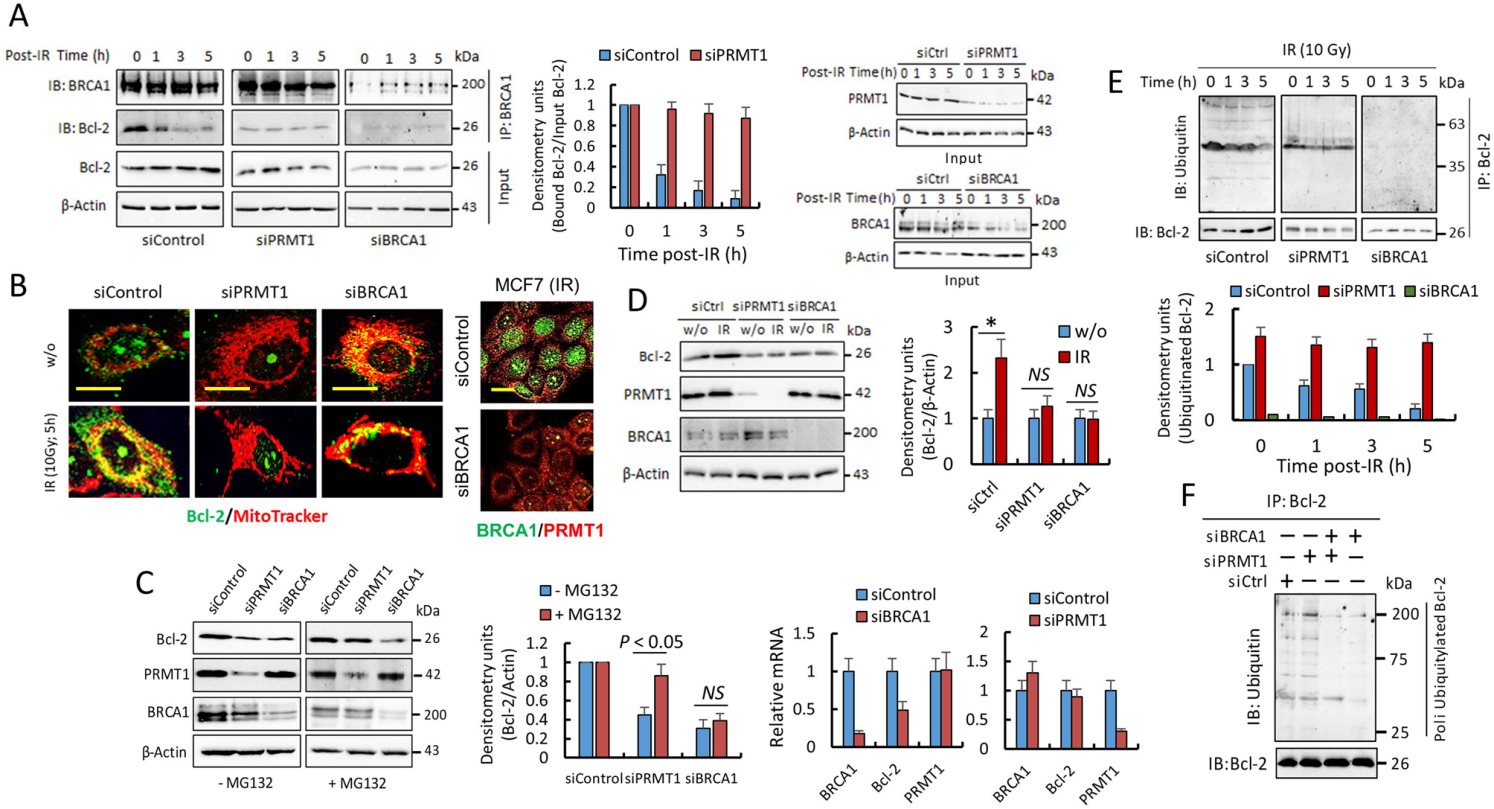
PRMT1-dependent methylation of BRCA1 controls the stability and localization of Bcl-2. (**A**) Coimmunoprecipitation of BRCA1 and Bcl-2 in MCF7 cells (IR, 10 Gy). Total extracts (1 mg) were immunoprecipitated using anti-BRCA1 (clone 6B4). Samples from different experimental conditions were processed in parallel and subjected to the same exposition time and image conditions (Fig. S9). The histogram represents coimmunoprecipitated Bcl-2 with respect to input Bcl-2 in siControl and siPRMT1 cells and relative to nonirradiated cells. The IR-dependent decrease in Bcl-2 was only statistically significant in siControl cells (*P* < 0.005 at all assayed times). Effective silencing was determined in the inputs (right panels). (**B**) Confocal microscopy showing Bcl-2 localization in MCF7 cells. Mitochondria were stained with MitoTracker-Red CMXRos. Bar represents 5 μm. BRCA1 silencing was evaluated (right panel; bars, 15 μm). (**C**) The effects of PRMT1 or BRCA1 silencing on Bcl-2 protein and mRNA levels in MCF7 cells in the absence or the presence of MG132 (10 µM; 10 h). Decrease of Bcl-2 mRNA was only significant in siBRCA1 cells when compared with siControl cells. (**D**) Effect of IR (10 Gy; 5 h) on Bcl-2 protein levels in the indicated MCF7 cells (*P < 0.05). (**E**) The ubiquitinated state of Bcl-2 in MCF7. Samples from different experimental conditions were processed in parallel and subjected to the same exposition time and image conditions (Fig. S9). The histograms represent ubiquitinated Bcl-2 with respect to immunoprecipitated Bcl-2 and relative to ubiquitinated Bcl-2 in nonirradiated siControl cells. The IR-dependent decrease in ubiquitinated Bcl-2 was only statistically significant in siControl cells (*P* < 0.005 at all assayed times). Silencing of PRMT1 or BRCA1 was tested in the inputs and results were similar as observed in **A**. (**F**) Western blot showing poly-ubiquitylated forms of Bcl-2 in MCF7 cells after indicated siRNA transfections. Equal amounts of Bcl-2 per lane were loaded. Silencing of PRMT1 and/or BRCA1 was tested in the inputs (similar results as observed in Fig. 5A). The groupings blots in this figure were cropped from different gels. Full blots are shown in the Supplementary Information, Fig. S9.

Taken together, these results confirm the previous observation that DNA damage-induced cytotoxicity is dissociated from the DNA repair function of BRCA1 but is dependent on BRCA1 cytosolic accumulation^13^. Since the proapoptotic capacity of BRCA1 has been attributed to its possible relationship with the antiapoptotic protein Bcl-2^14^ here, we analyzed the role of PRMT1 in the interaction of BRCA1 and Bcl-2 after irradiation. Immunoprecipitation assays indicated that under resting conditions, BRCA1 interacts with Bcl-2 in MCF7 cells; however, both proteins dissociate after IR (Fig. 6A). Interestingly, after irradiation and coinciding with BRCA1/Bcl-2 complex dissociation, Bcl-2 was clearly located in mitochondria in MCF7 cells (Fig. 6B). PRMT1 highly influenced the BRCA1/Bcl-2 interaction in these cells because silencing of this methylase clearly prevented the dissociation of BRCA1 and Bcl-2 after IR (Fig. 6A), and consequently, Bcl-2 was not found in mitochondria after IR exposure (Fig. 6B). As a control experiment, and as expected, silencing BRCA1 did not result in BRCA1/Bcl-2 complex formation (Fig. 6A), but interestingly, under BRCA1 depletion conditions, Bcl-2 was found to be constitutively localized in mitochondria in both irradiated and nonirradiated cells (Fig. 6B). In agreement with these results, we also observed preferential location of Bcl-2 in mitochondria in MDA-MB-436 cells, a breast cancer cell line highly resistant to IR-induced apoptosis (Supplementary Fig. S4). MDA-MB-436 cells have a BRCA15396+1G>A mutation that results in loss of BRCA1 protein expression^25^, deriving in constitutive DNA damage even in the absence of IR (Supplementary Fig. S4). As expected, irradiation of MDA-MB-436 cells resulted in undetectable BRCA1 foci and an increase in cumulative DNA DSB damage (Supplementary Fig. S4).

On the other hand, silencing experiments also indicated that both BRCA1 and PRMT1 modulated the intracellular levels of Bcl-2 in MCF7 cells (Fig. 6C). MCF7 cells depleted of PRMT1 or BRCA1 showed a significant decrease in Bcl-2 protein, but experiments in the presence of MG132 (Fig. 6C), a proteasome inhibitor, and analysis of Bcl-2-mRNA (Fig. 6C) indicated that BRCA1 and PRMT1 might differentially regulate Bcl-2 protein levels in breast cancer cells. Thus, although BRCA1 seems to be involved in the regulation of Bcl-2 expression^26^, PRMT1 is mainly implicated in Bcl-2 protein stability. In agreement with the transcriptional involvement of BRCA1 in Bcl-2 expression, we observed a significant increase in Bcl-2 protein after irradiation of siControl-MCF7 cells; however, depletion of BRCA1 or PRMT1 in these cells, which differentially inhibit the transcriptional functions^11^ of BRCA1, greatly avoided the IR-dependent overexpression of Bcl-2 (Fig. 6D).

Since the interaction of BRCA1 with Bcl-2 seems to be involved in Bcl-2 stability and BRCA1 has an E3 ubiquitin ligase activity^27^, we next analyzed the ubiquitination state of Bcl-2 under several experimental conditions (Fig. 6E,F). Interestingly, the level of Bcl-2 ubiquitination was clearly related to its interaction with BRCA1. Thus, irradiation of siControl-MCF7 cells showed a time-dependent decrease in Bcl-2 ubiquitination (Fig. 6E) coinciding with the dissociation of the BRCA1/Bcl-2 complex (Fig. 6A). However, in cells depleted of PRMT1, in which a continuous association of BRCA1 and Bcl-2 was observed (Fig. 6A), ubiquitination of Bcl-2 was found independent of irradiation (Fig. 6E). The decrease of the poly-ubiquitylated forms of Bcl-2 in cells depleted of BRCA1, independently of PRMT1 expression (Fig. 6E,F), also confirmed that cytosolic BRCA1 might control the stability of Bcl-2 in breast cancer cells. Whether ubiquitination of Bcl-2 by BRCA1 is a sufficient signal for its direct destruction in the proteasome or whether it mediates the degradation mechanism of Bcl-2 by XIAP and ARTS^28^ is still an issue that needs to be resolved.

## Discussion

Radiotherapy of tumors operates by inducing DSBs in cancer cells; however, IR-resistant cells rapidly initiate mechanisms to repair damage, enabling survival. While the DNA repair mechanisms responsible for cancer cell survival following DNA damaging treatments are well understood, less is known about the epigenetic mechanisms resulting in resistance to radiotherapy. DNA methylation changes in response to radiotherapy have been observed in MDA-MB-231 breast cancer cells, and many of these hyper- or hypomethylated genes following radiotherapy have relevant functions in DNA repair, cell cycle regulation and apoptosis^29^. However, prevalent changes in the epigenome require an operative cell division machinery to transmit these methylation patterns to daughter cells. Since cells exposed to IR subsequently arrest in various phases of the cell cycle to repair damaged DNA, these changes in DNA methylation could be more related to mechanisms of long-term resistance that allow the acquisition of resistant phenotypes. For instance, sublethal doses of radiation are sufficient to induce senescence in cancer cells, forcing them to acquire several new properties that are related to cell cycle progression and with the acquisition of stem cell characteristics^30^. With this in mind, it would be more likely that the methylation of a series of proteins (histones and non-histones) could be responsible for carrying out the first line of epigenetic defense in cells after IR. Therefore, understanding the epigenetic mechanisms that control the methylation of proteins responsible for DNA repair after IR (including BRCA1) could be important to design therapies that avoid the resistance of tumor cells to radiotherapy.

Epigenetics and metabolism are highly and reciprocally interconnected^31,32^. In addition to the modifications of their expression levels, other metabolic factors might control the intracellular activity of cellular methylases. For example, changes in the intracellular concentrations of their substrates could be an attractive mechanism of methylase regulation. Thus, metabolic changes that produce fluctuations in the intracellular levels of SAM could act as metabolic signals to turn on or off the epigenetic programs within the cell. An elegant demonstration of this regulatory mechanism during osteoclast differentiation has recently been reported^16^. The authors found that receptor activator of nuclear factor-κB ligand (RANKL), an essential cytokine for osteoclastogenesis, induces a metabolic shift toward oxidative metabolism, which is accompanied by an increase in SAM production. This overproduction of SAM, consequently, resulted in DNA methylation by DNA methyltransferase 3a and repression of anti-osteoclastogenic genes^16^. Here, we report a mechanism by which irradiation of breast cancer cells leads to increased SAM production.

Although an increase in SAM production after IR will surely affect the activity of multiple methylases within cells, as observed for the activation of PP2A, here we concentrate on the epigenetic regulation of BRCA1, a tumor suppressor protein involved in DNA repair, apoptosis and cell cycle regulation and highly involved in the resistance of breast cancer cells to radiotherapy. Here, we observed that the localization of BRCA1 and, therefore, its cellular functions are epigenetically controlled by PRMT1. From the first evidence that suggested that arginine methylation regulates the function of the nuclear import signal^33^, the nuclear localization of other transcription factors, such as FOXO1, has been reported to be mediated by PRMT1^7^. BRCA1 localization has an important role in the functions of this protein, which has both cytoplasmic and nuclear targets. However, the mechanisms underlying this shuttling process are not clearly understood. In addition to methylation, other post-translational modifications, such as phosphorylation of Thr508 by Akt, have also been reported to result in cytoplasmic accumulation of BRCA1^34^. Whether methylation/phosphorylation of nuclear localization signal (NLS) regions are involved in the regulation of shuttling mechanisms of BRCA1, similar to what happens with the FOXO1 transcription factor^7^, is a relevant issue that should be addressed. If this were the case, it would be tempting to speculate that other processes associated with the production of SAM could also modulate the cellular location of BRCA1. Since PP2A is now believed to dephosphorylate Akt at the Thr308 phosphoinositide-dependent kinase-1 (PDK1) phosphorylation site^35^, an attractive hypothesis that may link metabolic SAM overproduction with BRCA1 regulation is that irradiation may also inactivate Akt cellular functions. Therefore, Akt/PP2A and BRCA1/PRMT1 could be part of a cascade of epigenetic signaling that, through changes in intracellular SAM levels, would allow rapid activation of BRCA1 in response to DNA damage. However, on the other hand, PP2A has been described as a negative regulator of PRMT1 in a hepatocyte-derived carcinoma cell line^36,37^, taking these data into consideration, new studies should be performed to elucidate the involvement of PP2A on the methylation of BRCA1 after IR in breast cancer and other tumor cell types, since it might be cell-dependent.

**Figure 7:**
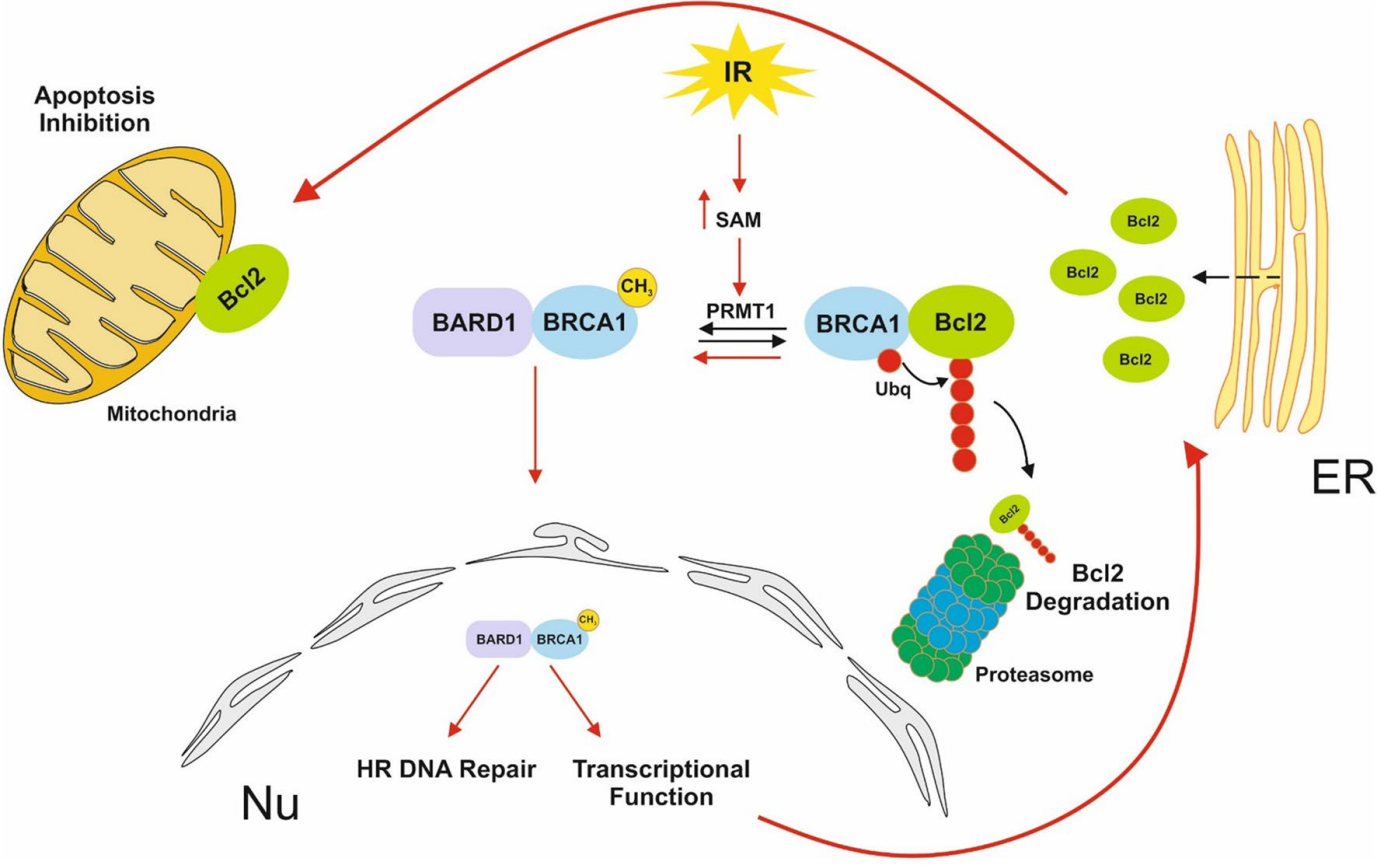
Epigenetic control of BRCA1 in breast cancer cells. Lack of BRCA1 results in constitutive DNA damage in breast cancer cells even in the absence of DNA-damaging agents. Since BRCA1 is found in the nucleus (Nu) of breast cancer cells (in addition to other cytoplasmic organelles, such as endoplasmic reticulum; ER)^14^ under normal growing conditions, the PRMT1-dependent methylation of BRCA1 might represent a constitutive mechanism to maintain DNA integrity under normal cell division. However, under IR stress (red arrows), overproduction of SAM induces massive BRCA1 methylation, which results in cytosolic BRCA1 depletion and its accumulation in the nucleus (after association with BARD1). Once in the nucleus, BRCA1 mediates HR DNA repair and carries out its transcriptional functions, including the transcription of the antiapoptotic protein Bcl-2. Under these conditions, the absence of BRCA1 in the cytosol prevents the degradation of newly synthetized Bcl-2, which is translocated to mitochondria to exert its antiapoptotic functions. In this scenario, PRMT1 becomes an important regulator of the oncogenic functions of BRCA1. Depletion of PRMT1 in irradiated breast cancer cells induces a change in the nuclear/cytoplasmic BRCA1 ratio, resulting in a switch in BRCA1 functions from repair and survival in the nucleus to activation of cell death signals in the cytoplasm. Thus, the consequent lack of nuclear BRCA1 in PRMT1-depleted cells allows massive DNA damage while activating apoptosis by complementary mechanisms. First, cells cannot synthetize novel Bcl-2, and second, cytosolic BRCA1 guides the continuous degradation of Bcl-2, which results in a total loss of cellular Bcl-2 and, consequently, the induction of apoptosis. This mechanism not only explains why BRCA1 has a proapoptotic function when cytosolic^13^ but also suggests that the unrepaired DSBs alone resulting from the loss of BRCA1 repair function might not be sufficient to fully affect the cytotoxicity and sensitivity of these cells to DNA-damaging agents. The cytosolic location of BRCA1 following DNA damage has been proposed as the process that links failed repair of DNA damage to the induction and execution of cell death processes^13^, and here, our results clearly demonstrate that this process is controlled epigenetically.

In addition to DNA repair, BRCA1 also plays a role in apoptosis^13,14^. When in the nucleus, BRCA1 controls high fidelity repair of damaged DNA, but in contrast, BRCA1 has been shown to enhance p53-independent apoptosis when cytoplasmic. The relationship between BRCA1 and the antiapoptotic Bcl-2 protein could explain the involvement of BRCA1 in apoptosis. Thus, cytosolic BRCA1 promotes Bcl-2-proteasome-dependent degradation, while nuclear BRCA1 activates the synthesis of Bcl-2. We observed that IR-dependent translocation of BRCA1 to the nucleus avoids cytosolic BRCA1-Bcl-2 association, which results in Bcl-2 stabilization and its accumulation in mitochondria. Altogether, the results indicate that under cytotoxic stimuli, such as IR, translocation of BRCA1 to the nucleus becomes essential to avoid IR-dependent apoptosis (Fig. 7). The proposed mechanism also explains why cytosolic BRCA1 has been found to be necessary for apoptosis induction under DNA damage stress; depletion of BRCA1 promotes Bcl-2 mitochondrial proapoptotic functions while inhibiting efficient DNA HR repair. In the absence of BRCA1 (or in cells with a dysfunctional BRCA1 protein), HR is suppressed, and the error-prone NHEJ takes over, which may lead to the accumulation of chromosome aberrations and eventually can lead to more tumorigenic cells^24^. Interestingly, we found that all these processes that lead to nuclear translocation of BRCA1 and apoptosis inhibition after irradiation are epigenetically controlled by PRMT1.

Since PRMT1-dependent methylation of BRCA1 contributes to the epigenetic defense of breast cancer cells to IR (and probably to other DNA damage agents), these could explain, at least in part, why PRMT1 has been identified as a marker of unfavorable prognosis for breast cancer patients^38^. On the other hand, the results also indicate that precise regulation of BRCA1 functions to avoid DNA repair in the nucleus but activate cell death signals in the cytoplasm might represent an attractive strategy for combined breast cancer therapies. Here, the results highlight the potential of PRMT1 as a clinical molecular target to modulate BRCA1 functions after IR and suggest that pharmacological inhibition of this protein or the use of hypomethylating treatments could be therapeutically useful for sensitizing breast tumors to radiotherapy^12^.

## Methods

### Reagents and antibodies

AA, AdOx, quinacrine, monensin, MG132, and AZA were obtained from Merck (Madrid, Spain). Antibodies used in this study are listed in Supplementary information Methods.

### Cell lines, treatments, and apoptosis assays

The MCF7, MCF7-Luc2, MDA-MB-436, and MDA-MB-231 human breast cancer cell lines and the murine cell line 4T1 were purchased from American Type Culture Collection (ATCC, Manassas, VA, USA). Human cells were routinely authenticated using genotype profiling according to the ATCC guidelines. Cells were maintained in the appropriate culture medium supplemented with 10% fetal calf serum and antibiotics. Cell viability was evaluated using 3-(4,5-dimethylthiazol-2-yl)-2,5-diphenyl-tetrazolium bromide. When indicated, cells were treated with AA (100 nM) or AdOx (10 µM) 24 h prior to IR treatments. For IR assays, the cells were irradiated using an Andrex SMART 200E machine (YXLON International, Hamburg, Germany) operating at 200 kV and 4.5 mA with a focus-object distance of 20 cm at room temperature and at a dose rate of 2.5 Gy/min. The radiation doses were monitored using a UNIDOS universal dosimeter in a PTW Farmer ionization chamber TW 30010 (PTW-Freiburg, Freiburg, Germany) in a radiation cabin^12^. Apoptosis was analyzed as described by Montenegro et al*.*^12^ (Supplementary information Methods).

### CRISPR/Cas9 PRMT1 knockout

4T1-PRMT1-KO cells were generated using the CRISPR/Cas9 technique. 4T1 cells were grown to 60–70% confluency in a 6-well plate and transfected with the Santa Cruz Biotechnology (Dallas, TX, USA) plasmids PRMT1 CRISPR/Cas9 containing the specifics gRNA sequences and the Cas9 ribonuclease (PRMT1m sc-420952-KO-2) and the PRMT1 HDR plasmid containing the puromycin resistance gene for selection (PRMT1m sc-420952-HDR-2) using the FuGENE 6 transfection reagent and a 1:3 DNA/FuGENE 6 ratio. The transfection medium was left for 48 h. On day 2, the medium was replaced with fresh medium, and puromycin was added at 5 µg/ml. Cells were allowed to grow for three more days. After 5–6 days, single cell colonies were isolated and expanded. Positive clones were characterized by genomic DNA extraction followed by Sanger sequencing and western blot experiments. As a transfection control, 4T1 cells were exclusively transfected with the PRMT1 HDR plasmid and subjected to the same experimental procedures as 4T1-PRMT1-KO cells.

### Plasmid construction and transfections

The PRMT1 expression vector [PRMT1 (NM_001536) Human Tagged ORF Clone; CAT#: RC224239] was obtained from Origene (Rockville, MD, USA). pDEST-FRT/T0-Flag-BRCA1 was a gift from Daniel Durocher^39^ (Addgene plasmid#71117). Briefly, for transient transfections, indicated cells were seeded in 6-well plates at a density of 4 × 10^5^ cells/well. The following day, MCF7 or HEK293T cells were transfected with expression vectors (over-PRMT1 or over-BRCA1, respectively) or the empty vector (over-Control) using Lipofectamine 2000 (Thermo Fisher Scientific, Barcelona, Spain).

### Stealth RNA transfections

Specific Stealth siRNAs for PRMT1 (HSS142550) and BRCA1 (HSS101089) were obtained from Thermo Fisher Scientific and transfected into MCF7 and MDA-MD-231 cells using Lipofectamine 2000. Treatments were started at 48 h after siRNA transfection. Stealth RNA-negative control duplexes (Thermo Fisher Scientific) were used as control oligonucleotides, and the ability of the Stealth RNA oligonucleotides to knock down the expression of selected genes was analyzed using western blot analysis at 48 h after siRNA transfection.

### Immunoblotting and immunoprecipitation

Whole-cell lysates were collected by adding SDS-PAGE sample loading buffer. After sonication, the samples were boiled (10 min) and proteins were separated by SDS-PAGE and, then, they were transferred to nitrocellulose membranes and analyzed using immunoblotting (ECL Plus, GE Healthcare, Barcelona, Spain). For immunoprecipitation assays of cell extracts, the cells (~ 5 × 10^6^) were lysed in 500 μl of RIPA lysis buffer (50 mM Tris, pH 8.0, 150 mM NaCl, 1% NP40, 1% sodium deoxycholate, 0.1% SDS, 5 mM EDTA) supplemented with protease and phosphatase inhibitor cocktails (Merck) and 50 µM IOX1 (a histone demethylase inhibitor; Merck). The cell extracts were cleared by centrifugation (20,000×*g* for 15 min). The extracts were precleared in 30-min incubations with 20 μl of Pure Proteome Protein G Magnetic Beads (Merck) at 4 °C while being rotated. The antibodies (as indicated in the figure legends) were then added to the precleared extracts. After incubation for 1 h at 4 °C, 50 μl of Pure Proteome Protein G Magnetic Beads were added, and the extracts were further incubated for 20 min at 4 °C with rotation. After extensive washing, the bound proteins were analyzed using western blots. The unbound extracts were used as the positive inputs to determine protein loading. Cytosolic extracts were obtained using NE-PER Nuclear and Cytoplasmic Extraction Reagents (Thermo Fisher Scientific).

### Cell cycle analysis

Cell cycle analysis was performed as we have previously described^12^ (Supplementary information Methods).

### PCR analysis

mRNA extraction, cDNA synthesis, and conventional and quantitative real-time RT-PCR were performed under standard conditions^40^. Primers used in this study are listed in Supplementary information Methods.

### Evaluation of global DNA methylation status

DNA was obtained using a PureLink Genomic DNA Mini Kit (Invitrogen, Barcelona, Spain) according to the manufacturer’s protocol and quantified by measuring the absorbance at 260 nm (NanoDropH 1000, Thermo Scientific). DNA purity was confirmed by the ratio of absorbance at 260 nm and 280 nm, which was always greater than or equal to 1.8. A colorimetric assay (MethylFlash Methylated DNA 5-mC Quantification Kit, Epigenetic Group Inc., NY, USA) was used to determine the global DNA methylation levels.

### Metabolite measurements

Intracellular metabolites were isolated on ice by sonication of 10 × 10^6^ cells in 1 mL of ice-cold PBS using a 30 kHz sonicator with probe at 30% amplitude for three 20-s cycles with one minute breaks in between. The resultant cell-free supernatants were snap frozen and stored at − 80 °C. ATP in breast cancer cells was detected using the Luminescent ATP Detection Assay Kit (Abcam; Cambridge, UK; ab113849) according to the manufacturer’s protocol. Quantification of intracellular SAM and SAH concentrations was then conducted using the SAM and SAH ELISA Combo Kit from Cell Biolabs, Inc. (STA-671-C; San Diego, CA, USA) following the manufacturers’ protocol.

### PP2A assay and alkaline treatment of MCF7 cell extracts

PP2A assays were performed as we have previously described^41^ (Supplementary information Methods). Extracts (30 µl, containing 50 µg of protein) were mixed with the same volume of 0.2 M NaOH (pH 12.5) and incubated for 30 min at 42 °C. Samples were then concentrated and analyzed by SDS-PAGE followed by immunoblotting with an anti-PP2A antibody. As described previously^18^, alkaline treatment resulted in demethylation of PP2AC.

### Confocal microscopy

Laser-scanning confocal microscopy of fixed cells was performed using a Leica TCS 4D confocal microscope (Wetzlar, Germany) and as described previously^12,41^ (Supplementary information Methods).

### Athymic nu/nu mice

Female athymic nu/nu mice, 4–5 weeks of age (5 mice per group), were obtained from Harlan Laboratories (Indianapolis, IN, USA). They were exposed to a 12-h light/12-h dark cycle and had free access to a Harlan Teklad Rodent Diet 8604 (Harlan Teklad, Madison, WI, USA) and water. Mice were housed under aseptic conditions (positive air pressure in a designated mouse room with microisolator tops), and all mouse handling procedures were carried out under a laminar flow hood. After ~ 2 weeks of acclimatization after arrival, 4T1 and 4T1-KO-PRMT1 cells (5 × 10^5^ cells in 50 μl PBS) containing a luciferase reporter were subcutaneously injected into the left and right flanks of each animal as indicated in the corresponding Figs. Tumors were analyzed using the IVIS Imaging System (Caliper Life Sciences, Hopkinton, MA, USA). The indicated animals were subjected to radiotherapy starting on day 15 after the tumor cells were injected. Radiotherapy consisted, as we have previously described^12^, of a single 5-Gy dose that was delivered with the X-ray irradiator described above. Irradiation was locally confined to the tumors by shielding the rest of the body of the mouse with lead. Radiotherapy was performed on days 16, 18, 20, 24, 26 and 28 (a total of 30 Gy/mice) after tumor injection. We made every attempt to reach the conclusion using as small sample size as possible. We usually exclude samples if we observe any abnormality in terms of size, weight or apparent disease symptoms in mice before performing experiments. However, we did not exclude any animals here, as we did not observe any abnormalities in the present study. Neither randomization nor blinding was performed in this study.

### Image acquisition, quantification of western blots and statistical analysis

Western blot analyses and analyses of microscopy data were performed as we have previously described^12^. Analyses were repeated at least three times, with similar results. The results from one experiment are shown. To quantify the results, the western blots were scanned using a Bio-Rad ChemiDoc scanning densitometer (Bio-Rad Laboratories, Hercules, CA, USA). For other experiments, the mean ± SD of three measurements performed in triplicate were calculated. Numerical data were analyzed to determine statistical significance using Mann–Whitney tests for comparisons of means in the SPPS statistical software for Microsoft Windows, release 6.0 (Professional Statistic, Chicago, IL, USA). Individual comparisons were made using Student's two-tailed, unpaired *t* tests. The criterion for significance was *P* < 0.05 for all comparisons.

### Ethical statement

Animals were bred and maintained according to the Spanish legislation on the ‘Protection of Animals used for Experimental and other Scientific Purposes’ and in accordance with the directives of the European Community. All animal procedures were approved by the Ethical Committee of the University of Murcia and the Direccion General de Ganaderia y Pesca, Comunidad Autonoma de Murcia (Project reference A13151101).

## Supplementary information

Supplementary information.
