# Supplementary material for: PRMT1-dependent methylation of BRCA1 contributes to the epigenetic defense of breast cancer cells against ionizing radiation
Source: Sci Rep. 2020 Aug 6;10:13275. doi: 10.1038/s41598-020-70289-3 (PMC7413540; doi:10.1038/s41598-020-70289-3)

SUPPLEMENTAL INFO

PRMT1-dependent methylation of BRCA1 contributes to the epigenetic defense of breast cancer cells against ionizing radiation

María F. Montenegro, Rebeca González-Guerrero, Luis Sánchez-del-Campo, Antonio Piñero-Madrona, Juan Cabezas-Herrera, and José Neptuno Rodríguez-López

Supplementary information Methods

Antibodies: Antibodies against the following proteins were used: β-actin (Merck; monoclonal clone AC-15), ASYM24 (Merck; polyclonal), BARD1 (Abcam; polyclonal), Bcl-2 (Merck; monoclonal clone Bcl-2-100), BRCA1 (Abcam; monoclonal clone MS110), BRCA1 (Thermo Fisher Scientific; monoclonal clone 6B4), Flag (Merck; monoclonal clone M2), PP2A-C subunit (Merck; monoclonal clone 1D6), phospho-H2AX (Ser139; Merck; monoclonal clone JBW301), PRMT1 (Thermo Fisher Scientific; polyclonal), Methyl-PP2A-C subunit (Merck; monoclonal clone 2A10), SAM68 (Abcam; polyclonal), ubiquitin (Santa Cruz Biotechnology; polyclonal), and 53BP1 (Abcam; polyclonal).

Apoptosis Assays: Apoptosis was analyzed as described by Montenegro et al.[12] using an ELISA assay (Cell Death Detection ELISAPLUS, Roche Diagnostics, Barcelona, Spain) to detect mono- and oligonucleosomes in the cytoplasmic fractions of cell lysates using biotinylated anti-histone and peroxidase-coupled anti-DNA antibodies. The amount of nucleosomes was photometrically quantified at 405 nm by determining the peroxidase activity that was retained in the immunocomplexes. Apoptosis was defined as the specific enrichment of mono- and oligonucleosomes in the cytoplasm and calculated by dividing the absorbance of the treated samples by the absorbance of the untreated samples after correcting for the number of cells. The induction of apoptosis in MCF7 cells after 7 h of treatment with 2 μM staurosporine (100% apoptotic cells) was used to calculate the number of apoptotic cells. The Hoechst staining was also used to detect apoptosis on the basis of the methods of Montenegro et al.[12]. Replicate cultures of 1 $\times 10^5$ cells per well were plated in six-well plates. The cells were subjected to the specified treatments. After exchanging the medium for fresh medium, the cells were incubated with 5 µl of Hoechst 33342 solution (Merck) per well at 37°C for 10 min and then observed under a fluorescence microscope. Strong fluorescence was observed in the nuclei of apoptotic cells, whereas weak fluorescence was observed in non-apoptotic cells. Quantification of the apoptotic cells was performed by counting the cells in four random fields in each well.

Cell cycle analysis. MDA-MB-231 cells, after the indicated treatments, were resuspended in PBS and fixed in 70% ethanol–PBS for 30 min at 4°C. Fixed cells were washed with PBS and treated with RNase at 37°C for 30 min. Finally, the cells were stained with propidium iodide (PI) for 30 min at 37°C. Samples were analyzed using flow cytometry in a FACSort cytometer (Becton-Dickinson, Franklin Lakes, NJ, USA) and Cell Quest (BD Biosciences, San Jose, CA, USA) and ModFIT software (Verity Software House, Topsham, ME, USA)[12].

PCR analysis. Primers were designed using Primer Express version 2.0 software and synthesized by Thermo Fisher Scientific. The following primers for human genes were used: β-actin (forward: 5'-AGA AAA TCT GGC ACC ACA CC-3'; reverse: 5'-GGG GTG TTG AAG GTC TCA AA-3'), PRMT1 (forward: 5'-GAG GCG GTG GCA GAC TAG-3'; reverse: 5'-GAC ACA TCG GTC AGA CCA G-3'), and Bcl-2 (forward: 5'-GGA TTG TGG CCT TCT TTG AG-3'; reverse: 5'-CCA AAC TGA GCA GAG TCT TC-3').

PP2A assay. After two washes with 0.9% NaCl, total cellular proteins were extracted in lysis buffer containing 50 mM Tris·HCl (pH 7.5), 250 mM NaCl, 3 mM EDTA, 3 mM EGTA, 1% Triton X-100, and 0.5% NP-40 without phosphatase inhibitors. Specific PP2A activity was measured using the PP2A Immunoprecipitation Phosphatase Assay Kit (Merck). All procedures were performed according to the manufacturer's protocol, and changes in absorbance were measured at 650 nm in a Spectra-MAX 250 (Molecular Devices, Sunnyvale, CA, USA) plate reader.

Confocal microscopy. For indirect immunofluorescence studies, preparations of cells on glass slides were fixed with cold acetone for 5 min and then washed with PBS. The cells were incubated with 3% bovine serum albumin (BSA) for 20 min and then with primary antibodies (diluted 1:200 in PBS containing 1% BSA) for 2 h at room temperature. The cells were washed three times in PBS and incubated for 1 h at room temperature with Alexa Fluor dyes (Life Technologies), which were used as the secondary antibodies. After 3 washes with PBS, the cells were incubated with 0.01% 4'-6-diamidino-2- phenylindole (DAPI; Merck) in water for 5 min. To determine antibody specificity, primary antibodies were replaced with specific IgGs (diluted 1:200) during immunofluorescence. Coverslips were permanently mounted to the slides using fluorescent mounting medium (DAKO, Carpinteria, CA, USA) and allowed to dry overnight in the dark. Colocalization analysis was performed as previously described[41] with the Co-localization Finder plugin of ImageJ-NIH and showed images of Alexa Fluor 633 (red) merged with Alexa Fluor 488 (green) secondary antibodies with the colocalized pixels in orange. This plugin provides the Pearson overlap coefficient (Rr) ranging from −1 to 1, where 1 represents perfect colocalization, 0 represents random colocalization, and −1 represents perfect exclusion (http://rsb.info.nih.gov/ij/plugins/colocalization-finder.html). When indicated, MitoTracker-Red CMXRos (Thermo Fisher Scientific, M7512) was added to the culture media at a final concentration of 50 nM. The cells were incubated under normal culture conditions for 30 min and then visualized by fluorescence microscopy.

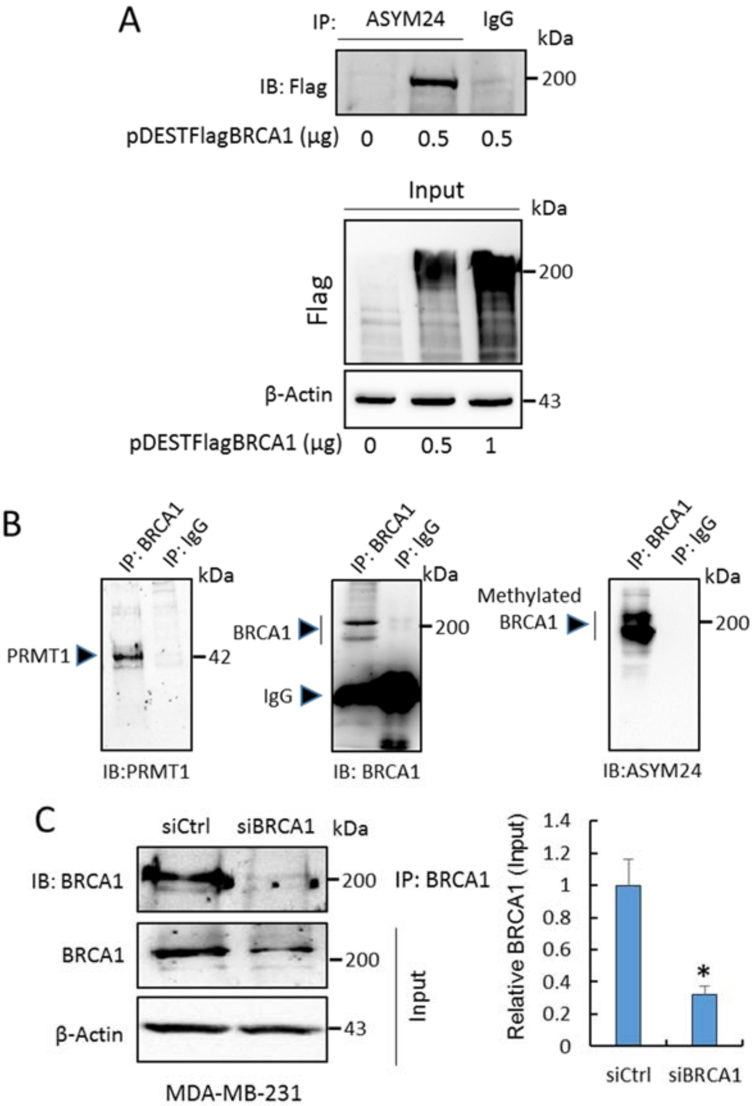

Supplementary Fig. S1. Specificity of the used antibodies. (A) Assays to prove anti-ASYM24 specificity. HEK293T cells were transfected with pDEST-FRT/T0-Flag-BRCA1. 48 h later, cells underwent immunoprecipitation with ASYM24 antibody or control IgG and immunoblotted with an anti-Flag antibody. (B) One milligram of MCF7 cytosolic extract was immunoprecipitated using an anti-BRCA1 antibody (clone 6B4), separated using SDS-PAGE and immunoblotted with the indicated antibodies. The negative controls were immunoprecipitated using an anti-IgG antibody, which did not result in representative bands when similar blotting conditions were used. Full westerns blots are presented. (C) Silencing of BRCA1 in MDA-MB-231 cells was used as a negative control to demonstrate antibody specificity. When cropped, the full blots are shown in the Supplementary Fig. S10.

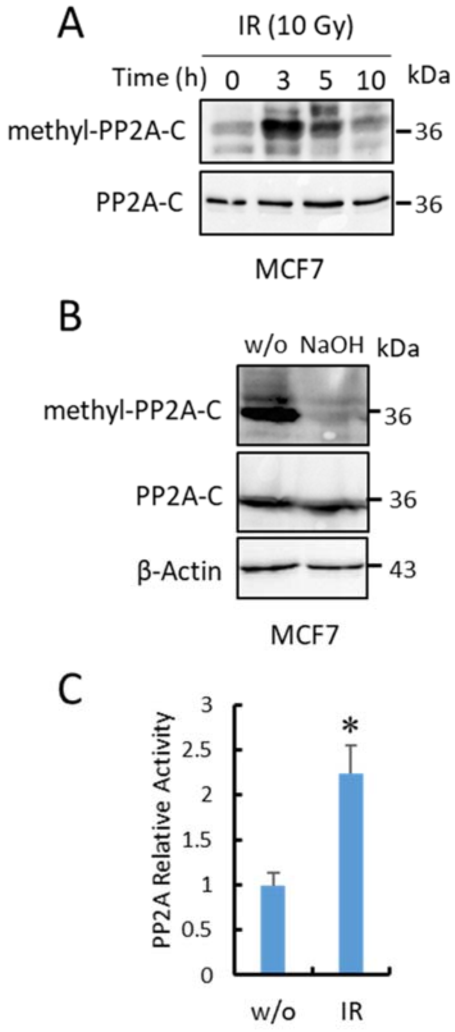

Supplementary Fig. S2. IR induces methylation of the catalytic PP2A C subunit and activates PP2A activity in MCF7 cells. (A) Effect of IR on the methylation status of the catalytic PP2A C subunit (Leu309) in MCF7 cells. The results are representative of three independent experiments. (B) Alkaline treatment resulted in demethylation of the PP2A C subunit; therefore, alkaline treatment was included to show the specificity of antibody labeling. (C) The histograms represent the effects of IR (10 Gy; 5 h) on PP2A activity. *$P < 0.05$ compared with untreated controls (w/o). The groupings blots in this figure were cropped from different gels. Full blots are shown in the Supplementary Fig. S11.

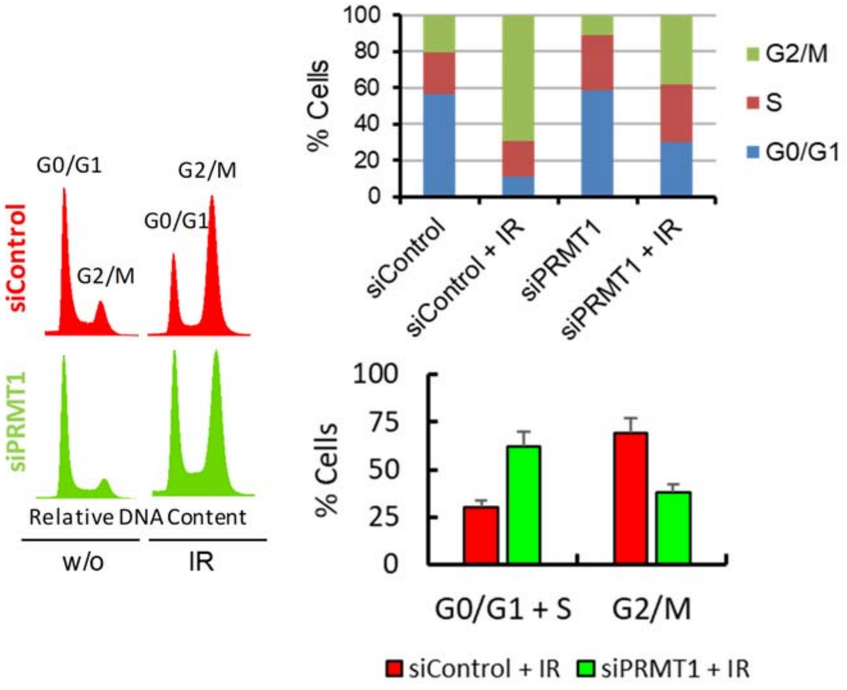

Supplementary Fig. S3. Cell cycle assays were performed using flow cytometry of MDA-MB-231 cells following the indicated treatments. After irradiation (5 Gy), cell cycle profiles were obtained, and analyses were performed at 24 h post irradiation. Assays were performed in triplicate, and differences in the cell cycle populations were found to be statistically significant ($P < 0.05$) when treated cells were compared with untreated control cells (w/o).

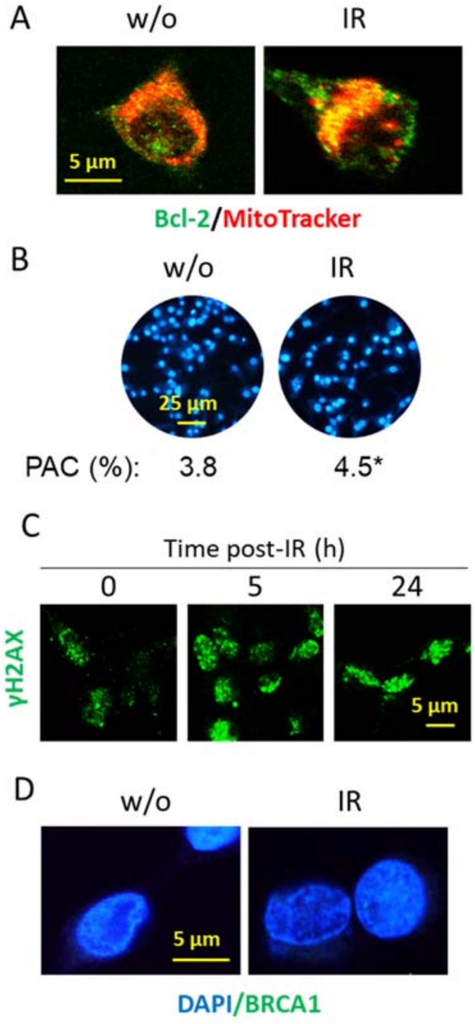

Supplementary Fig. S4. The effects of IR in MDA-MB-436 cells. (A) Confocal microscopy showing Bcl-2 localization in nonirradiated cells (w/o) and after IR (10 Gy; 5 h). Mitochondria were stained with MitoTracker-Red CMXRos. (B) Apoptosis was analyzed in control (w/o) and irradiated cells (IR; 10 Gy) using Hoechst's stain. Apoptosis was assayed on day 4 after IR. *PAC (Percentage of Apoptotic Cells) was not statistically significant when compared with nonirradiated cells. (C) Nuclear focus formation of γH2AX in nonirradiated (zero time) and irradiated (IR; 10 Gy) MDA-MB-436 cells. (D) Detection of BRCA1 and DAPI by immunofluorescence in nonirradiated cells (w/o) and after IR (10 Gy; 5 h).

Supplementary Fig. S5. The figure showed the full original and uncropped images for the western blots of Fig. 1 displayed in the text and results. The identification of the BRCA1, methylated BRCA1 (ASYM24), PRMT1, and β-actin bands was based on the expected molecular weight as indicated in the main figure.

Fig. 1A

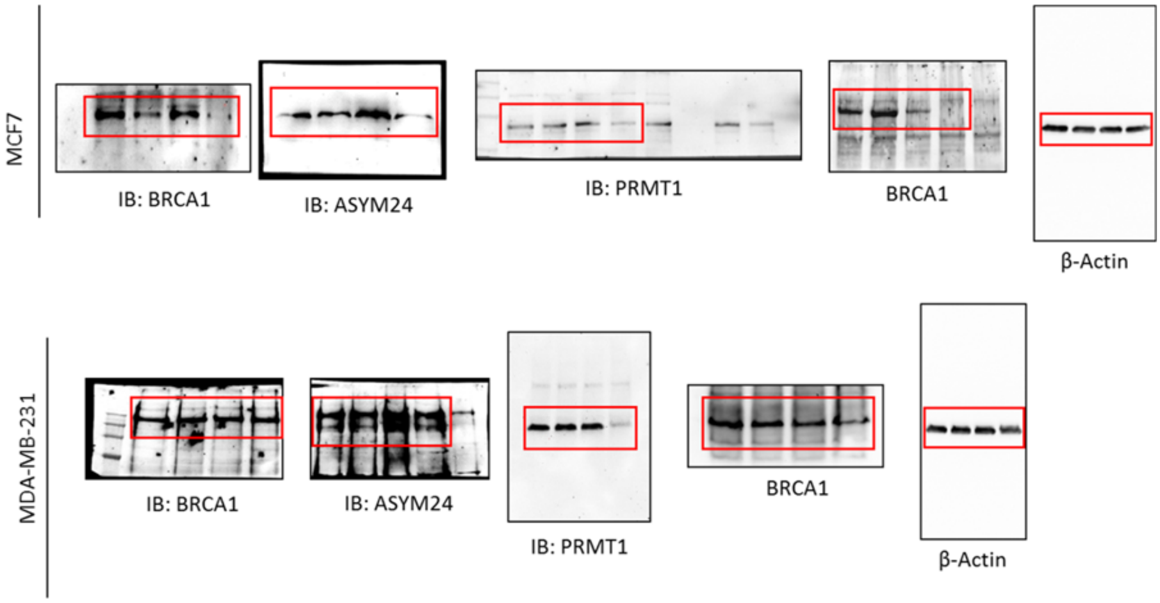

Fig. 1B

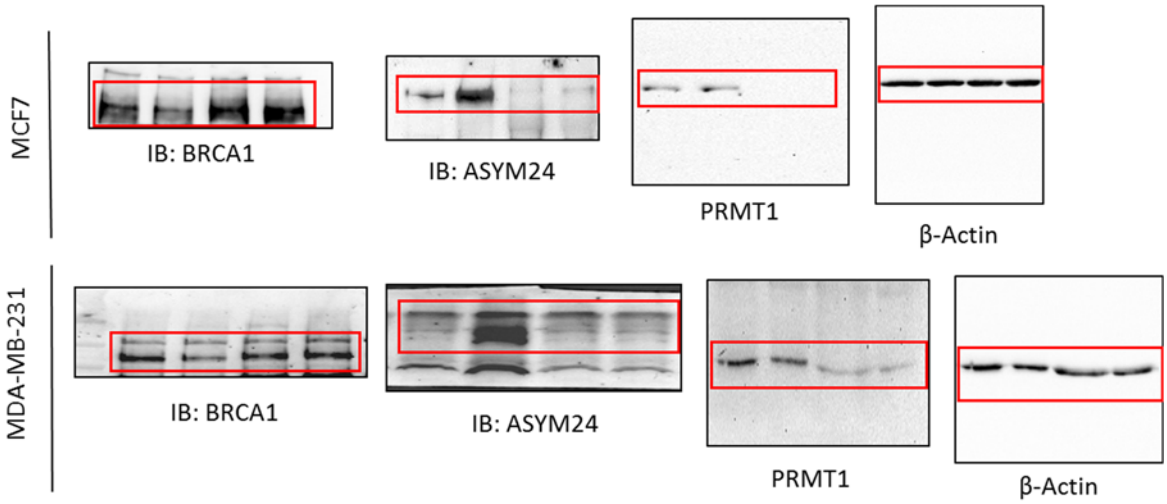

Fig. 1C

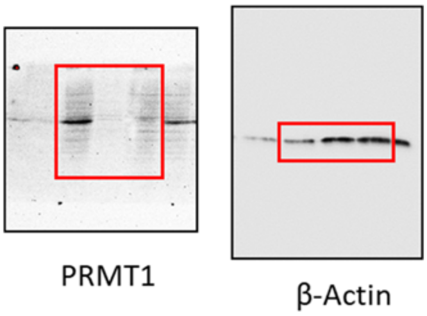

Fig. 1D

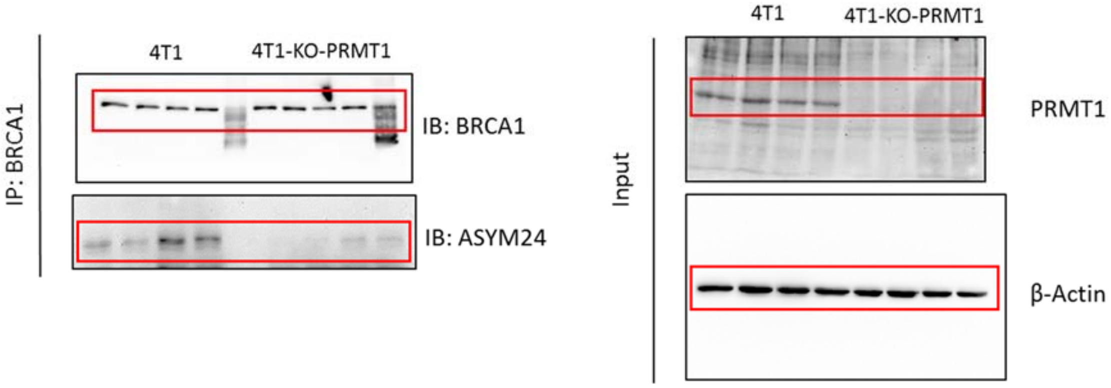

Fig. 1E

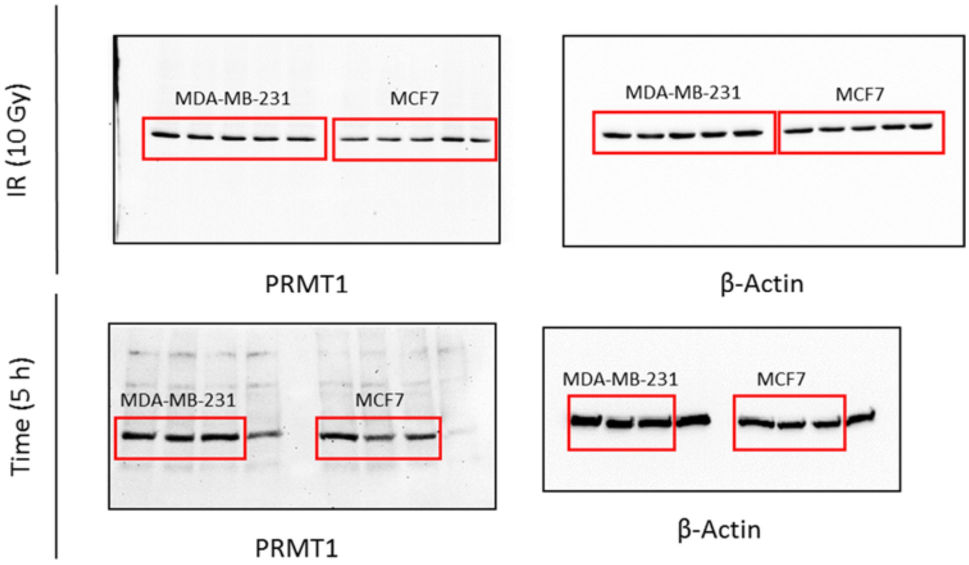

Supplementary Fig. S6. The figure showed the full original and uncropped images for the western blots of Fig. 3 displayed in the text and results. The identification of corresponding protein bands was based on the expected molecular weight as indicated in the main figure.

Fig. 3A

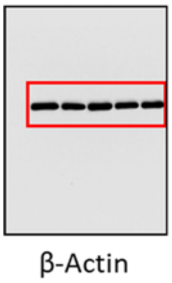

Fig. 3B

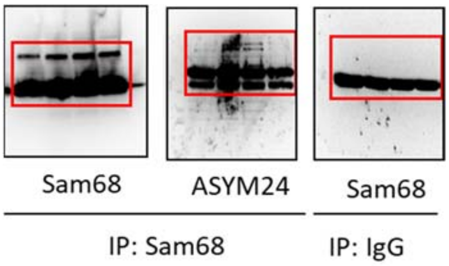

Fig. 3C

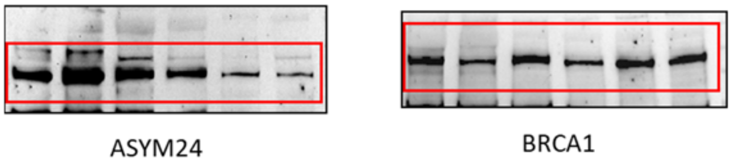

Supplementary Fig. S7. The figure showed the full original and uncropped images for the western blots of Fig. 4 displayed in the text and results. The identification of corresponding protein bands was based on the expected molecular weight as indicated in the main figure.

Fig. 4B

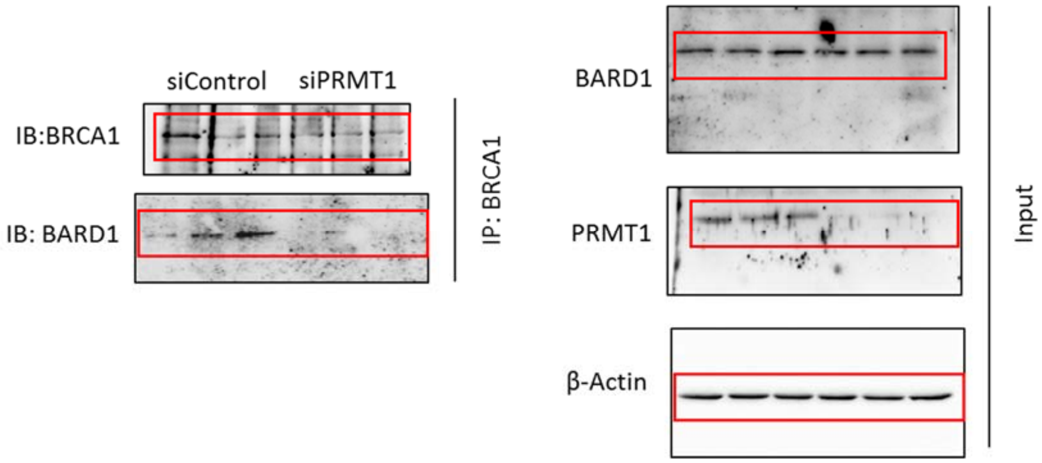

Fig. 4C

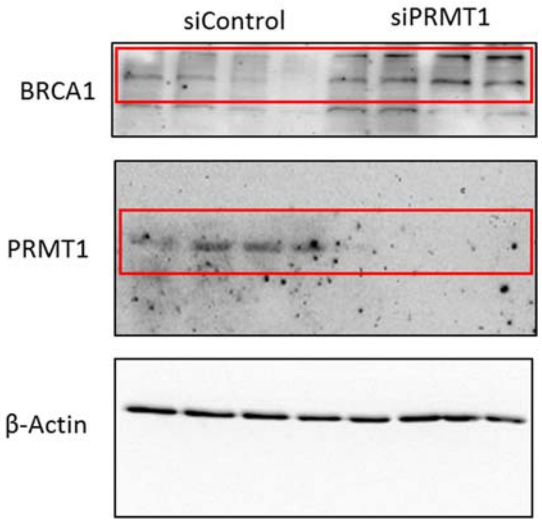

Supplementary Fig. S8. The figure showed the full original and uncropped images for the western blots of Fig. 5 displayed in the text and results. The identification of corresponding protein bands was based on the expected molecular weight as indicated in the main figure.

Fig. 5A

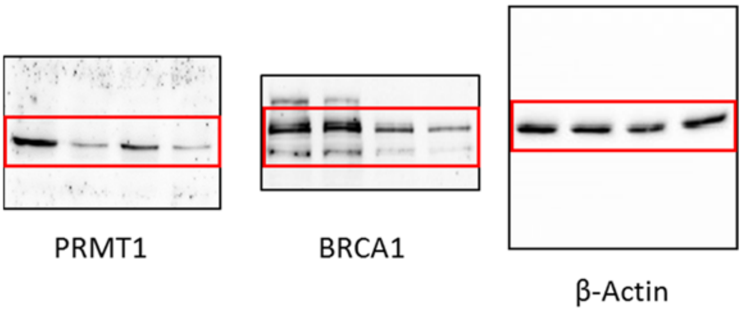

Fig. 5C

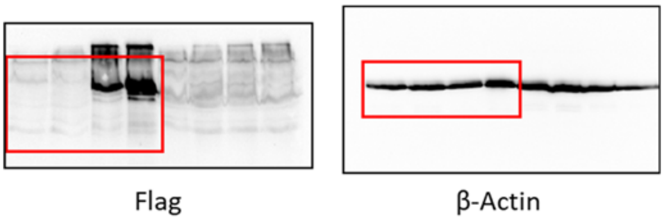

Supplementary Fig. S9. The figure showed the full original and uncropped images for the western blots of Fig. 6 displayed in the text and results. The identification of corresponding protein bands was based on the expected molecular weight as indicated in the main figure. Western blots for Bcl-2 (IB: Bcl-2; Fig. 6A) and Ubiquitin (IB: Ubiquitin; Fig. 6E) show images recorder at different Exposure times for comparison.

Fig. 6A

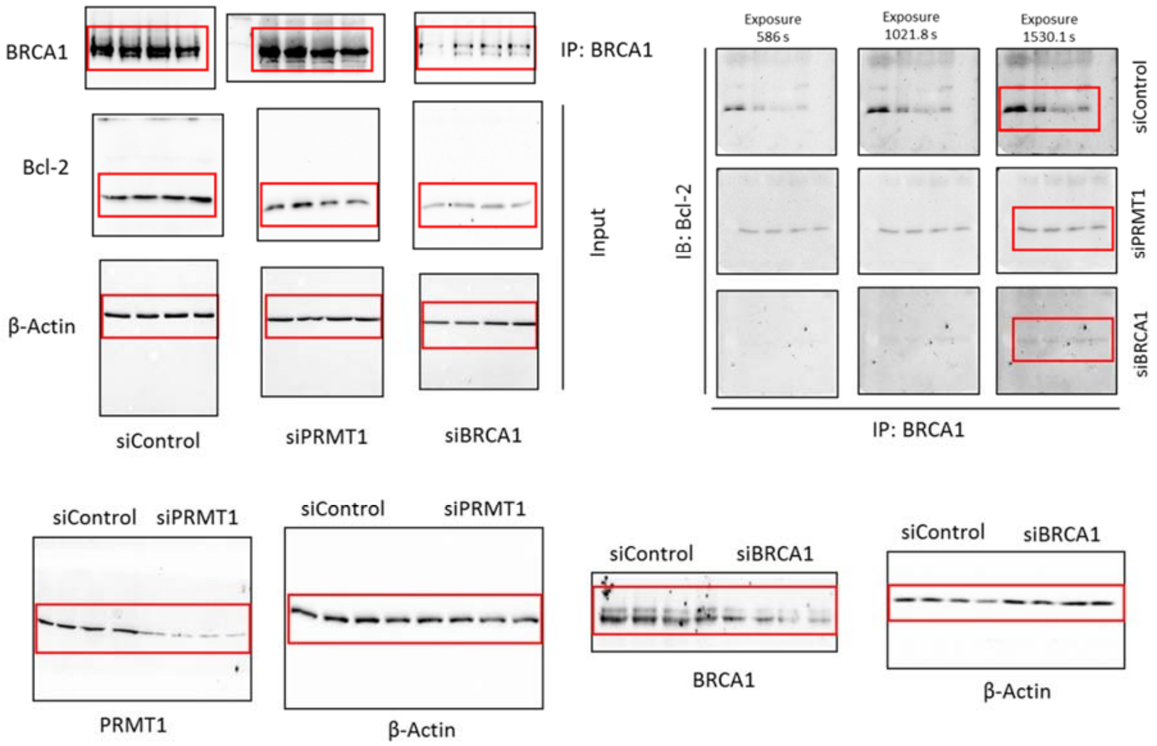

Fig. 6C

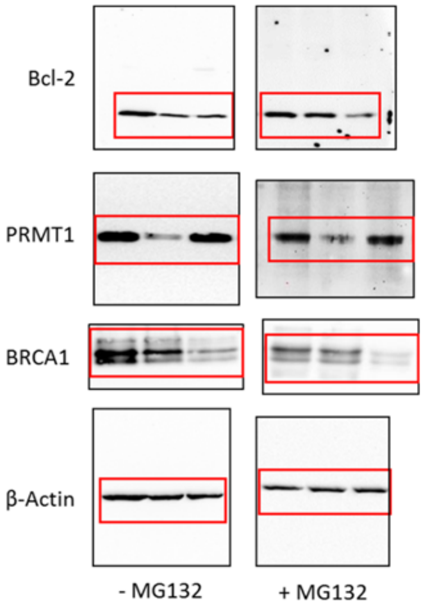

Fig. 6D

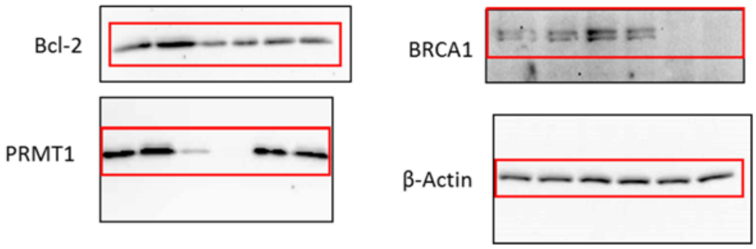

Fig. 6E

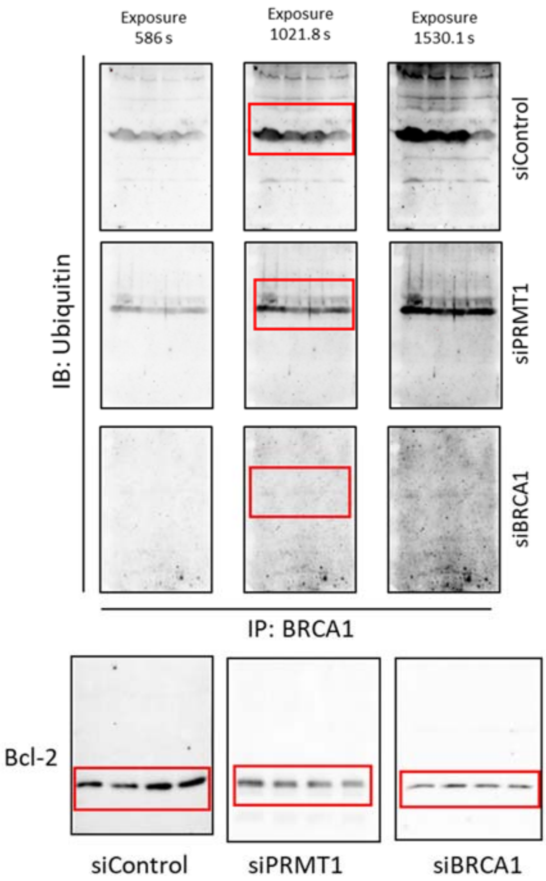

Fig. 6F

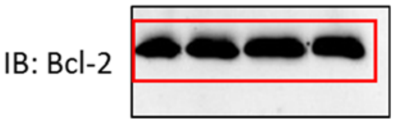

Supplementary Fig. S10. The figure showed the full original and uncropped images for the western blots of Supplementary Fig. S1. The identification of corresponding protein bands was based on the expected molecular weight as indicated in the main figure.

Supplementary Fig. S1A

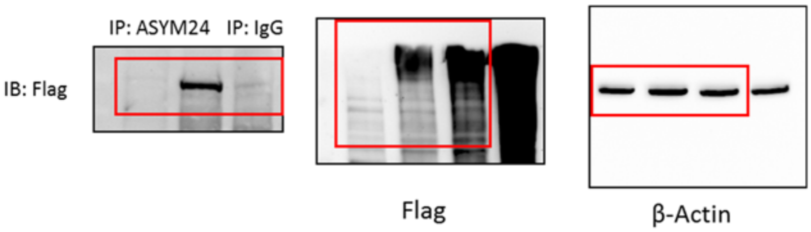

Supplementary Fig. S1C

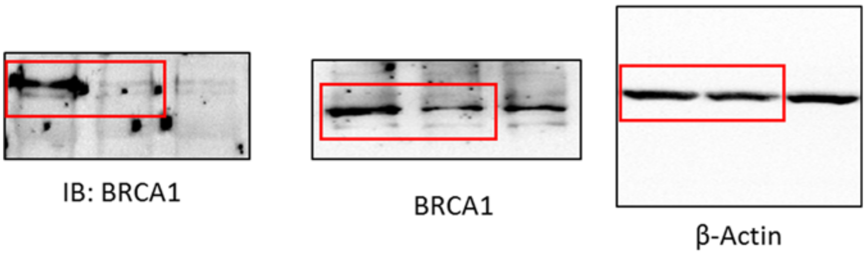

Supplementary Fig. S11. The figure showed the full original and uncropped images for the western blots of Supplementary Fig. S2. The identification of the PP2A-C, methylated PP2A-C, and β-actin bands was based on the expected molecular weight.

Supplementary Fig. S2A

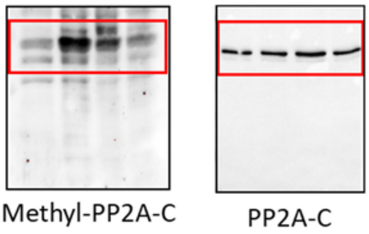

Supplementary Fig. S2B

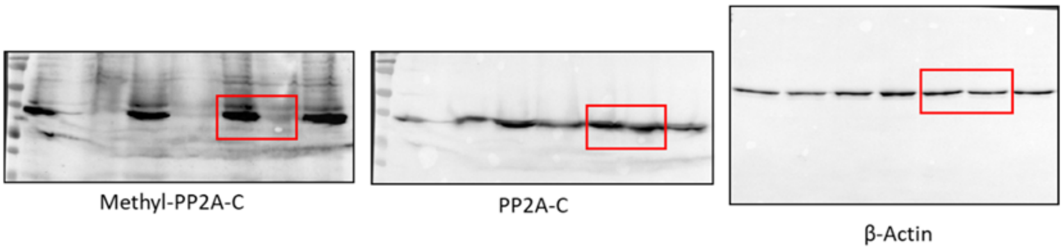

Supplement: Supplementary file 1 — Supplementary information. [file 41598_2020_70289_MOESM1_ESM.pdf]
